# Genome-wide association study of circulating interleukin 6 levels identifies novel loci

**DOI:** 10.1093/hmg/ddab023

**Published:** 2021-01-30

**Authors:** Tarunveer S Ahluwalia, Bram P Prins, Mohammadreza Abdollahi, Nicola J Armstrong, Stella Aslibekyan, Lisa Bain, Barbara Jefferis, Jens Baumert, Marian Beekman, Yoav Ben-Shlomo, Joshua C Bis, Braxton D Mitchell, Eco de Geus, Graciela E Delgado, Diana Marek, Joel Eriksson, Eero Kajantie, Stavroula Kanoni, John P Kemp, Chen Lu, Riccardo E Marioni, Stela McLachlan, Yuri Milaneschi, Ilja M Nolte, Alexandros M Petrelis, Eleonora Porcu, Maria Sabater-Lleal, Elnaz Naderi, Ilkka Seppälä, Tina Shah, Gaurav Singhal, Marie Standl, Alexander Teumer, Anbupalam Thalamuthu, Elisabeth Thiering, Stella Trompet, Christie M Ballantyne, Emelia J Benjamin, Juan P Casas, Catherine Toben, George Dedoussis, Joris Deelen, Peter Durda, Jorgen Engmann, Mary F Feitosa, Harald Grallert, Ann Hammarstedt, Sarah E Harris, Georg Homuth, Jouke-Jan Hottenga, Sirpa Jalkanen, Yalda Jamshidi, Magdalene C Jawahar, Tine Jess, Mika Kivimaki, Marcus E Kleber, Jari Lahti, Yongmei Liu, Pedro Marques-Vidal, Dan Mellström, Simon P Mooijaart, Martina Müller-Nurasyid, Brenda Penninx, Joana A Revez, Peter Rossing, Katri Räikkönen, Naveed Sattar, Hubert Scharnagl, Bengt Sennblad, Angela Silveira, Beate St Pourcain, Nicholas J Timpson, Julian Trollor, CHARGE Inflammation Working Group, Jenny van Dongen, Diana Van Heemst, Sophie Visvikis-Siest, Peter Vollenweider, Uwe Völker, Melanie Waldenberger, Gonneke Willemsen, Delilah Zabaneh, Richard W Morris, Donna K Arnett, Bernhard T Baune, Dorret I Boomsma, Yen-Pei C Chang, Ian J Deary, Panos Deloukas, Johan G Eriksson, David M Evans, Manuel A Ferreira, Tom Gaunt, Vilmundur Gudnason, Anders Hamsten, Joachim Heinrich, Aroon Hingorani, Steve E Humphries, J Wouter Jukema, Wolfgang Koenig, Meena Kumari, Zoltan Kutalik, Deborah A Lawlor, Terho Lehtimäki, Winfried März, Karen A Mather, Silvia Naitza, Matthias Nauck, Claes Ohlsson, Jackie F Price, Olli Raitakari, Ken Rice, Perminder S Sachdev, Eline Slagboom, Thorkild I A Sørensen, Tim Spector, David Stacey, Maria G Stathopoulou, Toshiko Tanaka, S Goya Wannamethee, Peter Whincup, Jerome I Rotter, Abbas Dehghan, Eric Boerwinkle, Bruce M Psaty, Harold Snieder, Behrooz Z Alizadeh

**Affiliations:** 1 Steno Diabetes Center Copenhagen, Gentofte DK2820, Denmark; 2 Department of Biology, The Bioinformatics Center, University of Copenhagen, Copenhagen DK2200, Denmark; 3 Department of Epidemiology, University of Groningen, University Medical Center Groningen, Groningen 9700 RB, The Netherlands; 4 Mathematics and Statistics, Murdoch University, Perth 6150, Australia; 5 Department of Epidemiology, University of Alabama at Birmingham School of Public Health, Birmingham, Alabama 35233, USA; 6 QIMR Berghofer Medical Research Institute, Brisbane 4006, Australia; 7 Department of Primary Care & Population Health, UCL Institute of Epidemiology & Health Care, University College London, London NW3 2PF, UK; 8 Institute of Epidemiology, Helmholtz Zentrum München—German Research Center for Environmental Health, Neuherberg 85764, Germany; 9 Department of Biomedical Data Sciences, Section of Molecular Epidemiology, Leiden University Medical Center, Leiden 2300 RC, The Netherlands; 10 Population Health Sciences, University of Bristol, Bristol BS8 2PS, UK; 11 Cardiovascular Health Research Unit, Department of Medicine, University of Washington, Seattle, WA 98101, USA; 12 Department of Medicine, University of Maryland School of Medicine, Baltimore, MD 21202, USA; 13 Department of Biological Psychology, Behaviour and Movement Sciences, Vrije Universiteit Amsterdam, Amsterdam 1081 BT, The Netherlands; 14 Amsterdam Public Health Research Institute, Amsterdam University Medical Center, Amsterdam 1105 AZ, The Netherlands; 15 Vth Department of Medicine (Nephrology, Hypertensiology, Rheumatology, Endocrinology, Diabetology), Medical Faculty Mannheim, University of Heidelberg, Mannheim 68167, Germany; 16 SIB Swiss Institute of Bioinformatics, Lausanne 1015, Switzerland; 17 Department of Internal Medicine and Clinical Nutrition, Sahlgrenska Academy, Centre for Bone and Arthritis Research (CBAR), University of Gothenburg, Gothenburg 41345, Sweden; 18 Chronic Disease Prevention Unit, National Institute for Health and Welfare, PO Box 30, Helsinki 00271, Finland; 19 Hospital for Children and Adolescents, Helsinki University Central Hospital and University of Helsinki, Helsinki 00014, Finland; 20 William Harvey Research Institute, Barts & the London Medical School, Queen Mary University of London, London EC1M 6BQ, UK; 21 The University of Queensland Diamantina Institute, The University of Queensland, Woolloongabba, Queensland 4102, Australia; 22 MRC Integrative Epidemiology Unit, Population Health Sciences, University of Bristol, Bristol BS8 2BN, UK; 23 Department of Biostatistics, Boston University School of Public Health, Boston, MA 02118, USA; 24 Centre for Genomic and Experimental Medicine, Institute of Genetics and Molecular Medicine, University of Edinburgh, Edinburgh EH4 2XU, UK; 25 Centre for Cognitive Ageing and Cognitive Epidemiology, University of Edinburgh, Edinburgh EH8 9JZ, UK; 26 Usher Institute, University of Edinburgh, Edinburgh EH8 9AG, UK; 27 Department of Psychiatry, Amsterdam UMC, Vrije Universiteit, Amsterdam 1081 HJ, The Netherlands; 28 Université de Lorraine, Inserm, IGE-PCV, Nancy 54000, France; 29 Istituto di Ricerca Genetica e Biomedica, Consiglio Nazionale delle Ricerche, Monserrato (CA) 09042, Italy; 30 Cardiovascular Medicine, Department of Medicine Solna, Center for Molecular Medicine, Karolinska Institutet, Stockholm 17176, Sweden; 31 Unit of Genomics of Complex Diseases, Institut d'Investigació Biomèdica Sant Pau (IIB-Sant Pau), Barcelona 08041, Spain; 32 Department of Clinical Chemistry, Fimlab Laboratories, and Finnish Cardiovascular Research Center—Tampere, Faculty of Medicine and Health Technology, Tampere University, Tampere 33520, Finland; 33 Institute of Cardiovascular Science, University College London, London WC1E 6BT, UK; 34 Discipline of Psychiatry, Adelaide Medical School, University of Adelaide, Adelaide 5005, Australia; 35 Institute for Community Medicine, University Medicine Greifswald, Greifswald 17475, Germany; 36 Centre for Healthy Brain Ageing, School of Psychiatry, University of New South Wales, Sydney 2052, Australia; 37 Division of Metabolic Diseases and Nutritional Medicine, Ludwig-Maximilians-University of Munich, Dr. von Hauner Children's Hospital, Munich 80337, Germany; 38 Department of Cardiology, Leiden University Medical Center, Leiden 2300 RC, The Netherlands; 39 Section of Gerontology and Geriatrics, Department of Internal Medicine, Leiden University Medical Center, Leiden 2333 ZA, The Netherlands; 40 Baylor College of Medicine, Houston, TX 77030, USA; 41 National Heart, Lung, and Blood Institute's and Boston University's Framingham Heart Study, Framingham, MA 01702, USA; 42 Section of Cardiovascular Medicine and Preventive Medicine, Department of Medicine, Boston University School of Medicine, Boston, MA 02118, USA; 43 Massachusetts Veterans Epidemiology Research and Information Center (MAVERIC), VA Boston Healthcare System, Boston, MA 02130, USA; 44 ^44^Department of Nutrition-Dietetics, Harokopio University, Athens 17671, Greece; 45 Max Planck Institute for Biology of Ageing, Cologne 50931, Germany; 46 Department of Pathology and Laboratory Medicine, Larner College of Medicine, University of Vermont, Burlington, VT 05405, USA; 47 Division of Statistical Genomics, Department of Genetics, Washington University School of Medicine, St. Louis, MO 63110-1093, USA; 48 German Center for Diabetes Research (DZD), Neuherberg 85764, Germany; 49 The Lundberg Laboratory for Diabetes Research, Department of Molecular and Clinical Medicine, Sahlgrenska Academy at the University of Gothenburg, Gothenburg SE-41345, Sweden; 50 Department of Psychology, University of Edinburgh, Edinburgh EH8 9JZ, UK; 51 Interfaculty Institute for Genetics and Functional Genomics, University Medicine Greifswald, Greifswald 17475, Germany; 52 MediCity Research Laboratory, University of Turku, Turku 20520, Finland; 53 Department of Medical Microbiology and Immunology, University of Turku, Turku 20520, Finland; 54 Genetics Research Centre, Molecular and Clinical Sciences Institute, St George's University of London, London SW17 0RE, UK; 55 ^55^Department of Epidemiology Research, Statens Serum Institute, Copenhagen DK2300, Denmark; 56 Department of Epidemiology & Public Health, UCL Institute of Epidemiology & Health Care, University College London, London WC1E 7HB, UK; 57 Turku Institute for Advanced Studies, University of Turku, Turku 20014, Finland; 58 Department of Psychology and Logopedics, University of Helsinki, Helsinki 00014, Finland; 59 Department of Epidemiology and Prevention, Wake Forest School of Medicine, Winston-Salem, NC 27157, USA; 60 Department of Internal Medicine, Lausanne University Hospital (CHUV), Lausanne 1011, Switzerland; 61 University of Lausanne, Lausanne 1011, Switzerland; 62 IBE, Faculty of Medicine, Ludwig Maximilians University (LMU) Munich, Munich 81377, Germany; 63 Institute of Medical Biostatistics, Epidemiology and Informatics (IMBEI), University Medical Center, Johhanes Gutenberg University, Mainz 55101, Germany; 64 Department of Clinical Medicine, University of Copenhagen, Copenhagen DK2200, Denmark; 65 BHF Glasgow Cardiovascular Research Centre, Faculty of Medicine, Glasgow G12 8TA, UK; 66 ^66^Clinical Institute of Medical and Chemical Laboratory Diagnostics, Medical University of Graz, Graz 8036, Austria; 67 Department of Cell and Molecular Biology, National Bioinformatics Infrastructure Sweden, Science for Life Laboratory, Uppsala University, Uppsala 75124, Sweden; 68 Max Planck Institute for Psycholinguistics, Nijmegen XD 6525, The Netherlands; 69 Donders Institute for Brain, Cognition and Behaviour, Radboud University, Nijmegen 6525 AJ, The Netherlands; 70 Department of Developmental Disability Neuropsychiatry, School of Psychiatry, University of New South Wales, Sydney 2031, Australia; 71 Department of Genetics, Environment and Evolution, University College London Genetics Institute, London WC1E 6BT, UK; 72 Department of Population Health Sciences, Bristol Medical School, University of Bristol, Bristol BS8 1UD, UK; 73 Dean’s Office, College of Public Health, University of Kentucky, Lexington, KY 40536, USA; 74 Department of Psychiatry, Melbourne Medical School, The University of Melbourne, Parkville 3000, Australia; 75 Department of Psychiatry and Psychotherapy, University of Muenster, Muenster 48149, Germany; 76 The Florey Institute of Neuroscience and Mental Health, The University of Melbourne, Parkville 3000, Australia; 77 ^77^Centre for Genomic Health, Queen Mary University of London, London EC1M 6BQ, UK; 78 National Institute for Health and Welfare, University of Helsinki, Helsinki 00014, Finland; 79 Department of General Practice and Primary Health Care, University of Helsinki, Helsinki 00014, Finland; 80 MRC Integrative Epidemiology Unit at the University of Bristol, Bristol BS6 2BN, UK; 81 Population Health Science, Bristol Medical School, University of Bristol, Bristol BS8 2BN, UK; 82 Icelandic Heart Association, Kópavogur 201, Iceland; 83 Faculty of Medicine, University of Iceland, Reykjavik 101, Iceland; 84 Institute and Clinic for Occupational, Social and Environmental Medicine, University Hospital, LMU Munich, Munich 81377, Germany; 85 Allergy and Lung Health Unit, Melbourne School of Population and Global Health, The University of Melbourne, Melbourne 3010, Australia; 86 Durrer Center for Cardiogenetic Research, Amsterdam 1105 AZ, The Netherlands; 87 Deutsches Herzzentrum München, Technische Universität München, Munich 80636, Germany; 88 ^88^DZHK (German Centre for Cardiovascular Research), partner site Munich Heart Alliance, Munich 80336, Germany; 89 Institute of Epidemiology and Medical Biometry, University of Ulm, Ulm 89081, Germany; 90 Institute for Social and Economic Research, University of Essex, Colchester CO4 3SQ, Germany; 91 University Center for Primary Care and Public Health, University of Lausanne, Lausanne 1010, Switzerland; 92 SYNLAB Academy, SYNALB Holding Deutschland GmbH, Mannheim 68163, Germany; 93 Neuroscience Research Australia, Sydney 2031, Australia; 94 Institute of Clinical Chemistry and Laboratory Medicine, University Medicine Greifswald, Greifswald 17475, Germany; 95 DZHK (German Center for Cardiovascular Research), partner site Greifswald, Greifswald 17475, Germany; 96 Centre for Population Health Research, University of Turku, Turku University Hospital, Turku 20520, Finland; 97 Research Centre of Applied and Preventive Cardiovascular Medicine, University of Turku, Turku 20520, Finland; 98 Department of Clinical Physiology and Nuclear Medicine, Turku University Hospital, Turku 20014, Finland; 99 Department of Biostatistics, University of Washington, Seattle, WA 98195, USA; 100 Neuropsychiatric Institute, Prince of Wales Hospital, Sydney 2031, Australia; 101 Novo Nordisk Foundation Center For Basic Metabolic Research, Section of Metabolic Genetics, Faculty of Health and Medical Sciences, University of Copenhagen, Copenhagen DK2200, Denmark; 102 Department of Public Health, Section on Epidemiology, University of Copenhagen, Copenhagen DK1014, Denmark; 103 Department of Twin Research and Genetic Epidemiology, King's College London, London SE1 7EH, UK; 104 MRC/BHF Cardiovascular Epidemiology Unit, Department of Public Health and Primary Care, University of Cambridge, Cambridge CB1 8RN, UK; 105 Longitudinal Study Section, Translational Gerontology Branch, National Institute on Aging, Baltimore, MD 21224, USA; 106 Population Health Research Institute, St George’s, University of London, London SW17 0RE, UK; 107 Department of Pediatrics, The Institute for Translational Genomics and Population Sciences, The Lundquist Institute at Harbor-UCLA Medical Center, Torrance, CA 90502, USA; 108 Department of Epidemiology, Erasmus MC, Rotterdam 3000 CA, The Netherlands; 109 Human Genetics Center, School of Public Health, University of Texas Health Science Center at Houston, Houston, TX 77030, USA; 110 Human Genome Sequencing Center, Baylor College of Medicine, Houston, TX 77030, USA; 111 Departments of Epidemiology and Health Services, University of Washington, Seattle, WA 98101, USA

## Abstract

Interleukin 6 (IL-6) is a multifunctional cytokine with both pro- and anti-inflammatory properties with a heritability estimate of up to 61%. The circulating levels of IL-6 in blood have been associated with an increased risk of complex disease pathogenesis. We conducted a two-staged, discovery and replication meta genome-wide association study (GWAS) of circulating serum IL-6 levels comprising up to 67 428 (*n*_discovery_ = 52 654 and *n*_replication_ = 14 774) individuals of European ancestry. The inverse variance fixed effects based discovery meta-analysis, followed by replication led to the identification of two independent loci, *IL1F10/IL1RN* rs6734238 on chromosome (Chr) 2q14, (*P*_combined_ = 1.8 × 10^−11^), *HLA-DRB1/DRB5* rs660895 on Chr6p21 (*P*_combined_ = 1.5 × 10^−10^) in the combined meta-analyses of all samples. We also replicated the *IL6R* rs4537545 locus on Chr1q21 (*P*_combined_ = 1.2 × 10^−122^). Our study identifies novel loci for circulating IL-6 levels uncovering new immunological and inflammatory pathways that may influence IL-6 pathobiology.

## Introduction

Interleukin 6 (IL-6) is a multifunctional cytokine, which is involved in a wide range of immunomodulatory processes, from cellular migration and adhesion to proliferation and maturation (1,2). Interleukins are involved in immune cell differentiation and activation (3). IL-6 is synthesized by a variety of different immune cells such as monocytes (4), B cells (5) and T cells (6) and also non-immune cells such as epithelial and smooth muscle cells (7), adipocytes (8), endothelial cells (9) and osteoblasts (10).

Several factors have been implicated in circulating IL-6 levels. We have previously demonstrated that IL-6 levels decrease with age in children and increase with age in adults (11). Also, increased levels of IL-6 have been observed in various diseases, not surprisingly in autoimmune diseases such as rheumatoid arthritis (12) and systemic juvenile idiopathic arthritis (13), but also cardio-metabolic diseases like type 2 diabetes (14), heart failure, coronary heart disease (15) and atherosclerosis (16), as well in cancers (17), atopic dermatitis (18) and psychological disorders like depression (19). Due to its implications in the pathogenesis of different disorders, Il-6 has been used as an appropriate choice for drug targeting and used as a monitoring biomarker of disease progression and response to treatments (20). The most illustrious IL-6 inhibitor is tocilizumab (21), a monoclonal antibody binding the IL-6 receptor, which is already in use for treating patients with allergic asthma (22), and immune system disorders like rheumatoid arthritis (23) and systemic juvenile idiopathic arthritis (24), with high efficacy with some initial benefits towards respiratory illnesses like COVID-19 (25).

IL-6 baseline levels are heritable with estimates from twin studies ranging between 15 and 61% (26–29). However, efforts to identify genetic variants associated with levels of IL-6 constituted relatively small-scale GWAS (30–33) or sequencing-based candidate gene association studies (34). To date, variants in the IL-6 receptor gene (*IL-6R*) and the gene encoding histo-blood group ABO system transferase (*ABO*) have been identified as statistically significant for an association to IL6-levels. Also, the genetic risk score constructed of *IL-6* variants identified in the study by Shah and colleagues explained up to 2% of the variation in IL-6 levels (33), leaving a substantial part of its heritability unexplained. These seemingly sparse results and limited findings could be due to limitations in the study power caused by low sample size or a great inter-individual variability of IL-6 levels. One may speculate a substantial increase in the study size by increasing the number of participants, which would very likely lead to the identification of additional variants explaining IL-6 levels (35–37).

The current study is the (till date) largest meta GWAS study including 67 428 individuals of European ancestry to identify genetic variants explaining the levels of circulating IL-6 and to understand underlying genetic mechanisms implicated in the pathophysiology of this cytokine.

## Results

A total of 52 654 individuals of European descent from 26 cohorts were included in the discovery GWAS meta-analysis with up to 2 454 025 autosomal SNPs passing quality control. Four cohorts (ALSPAC, MONICA/KORA, NTR and SardiNIA) identified genome-wide significant associations in the *ABO* region, whereas none of the other 22 cohorts did, either individually or combined. These cohorts conditioned their results on their relevant top-SNP in *ABO*, the results of which were included in the discovery meta-analyses. The overall genomic control inflation factor (*λ*_GC_ after correction) at the discovery stage meta-analysis was 1.0.

**Figure 1:**
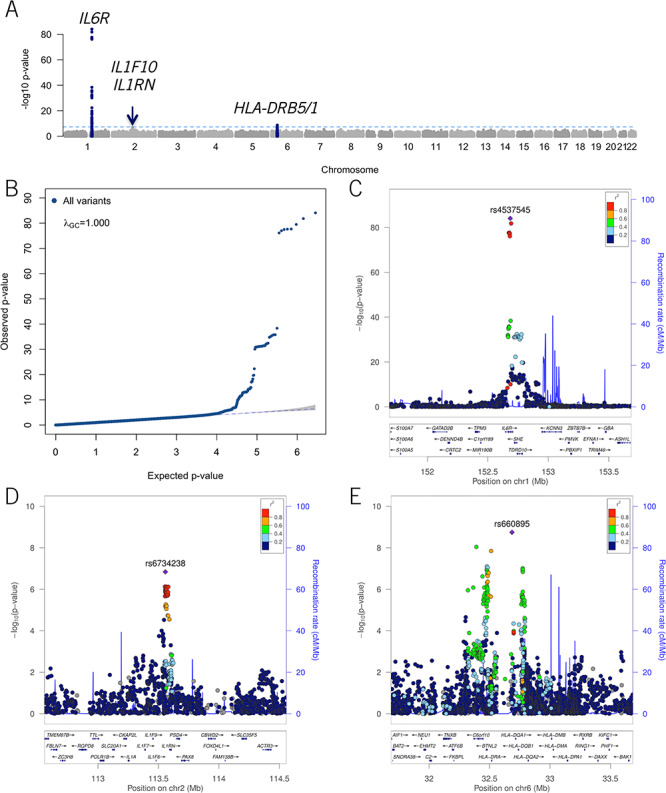
Manhattan, QQ and LocusZoom plots of the discovery GWAS meta-analyses. (**A**) Manhattan plot showing the association of SNPs with IL-6. Loci coloured in red or blue, three in total, represent those for which the lead SNPs reached genome-wide significance (*P* = 5 × 10^−8^). Horizontal axis: relative genomic position of variants on the genome, vertical axis: −log10 *P*-value of each SNP; (**B**) Quantile–quantile plot for *P*-values obtained from the meta-analysis. The horizontal and vertical axes represents the expected distribution of −log10 (*P*-values) under the null hypothesis of no association, whereas the vertical axis shows the observed −log10 (*P*-values). The blue dashed line represents the null, and *λ*_GC_ value represents the genomic inflation factor lambda. Each data point represents the observed versus the expected *P*-value of a variant included in the association analyses; (**C**–**E**) Regional association plots for each of the three genome-wide significant loci, 1q21, 2q14 and 6p21, respectively. Pairwise LD (*r*^2^) with the lead SNP is indicated following a color-coded scale. Horizontal axis: relative genomic position of variants within the locus, vertical axis: −log10 *P*-value of each SNP.

We identified 94 variants that were genome-wide significantly (*P*_discovery_ < 5.0 × 10^−8^; Supplementary Material, Table S1) associated with IL-6 levels, representing two independent genetic loci on chromosomes 1q21 and 6p21. Two common SNPs (rs4537545 and rs660895), one per locus, Chr. 1q21 (*IL6R*), and Chr. 6p21 (*HLA-DRB1*/*HLA-DRB5*), showed the most significant association with IL-6 levels (index SNPs) and the third SNP (rs6734238) mapped on Chr. 2q14 (*IL1F10*/*IL1RN*) locus showed suggestive (5.0 × 10^−8^ < *P*_discovery_ *<* 1.0 × 10^−5^) association in addition to 5 other loci (*LHFPL3*, *LZTS1*, *GPC5*/*GPC6*, *USP32*/*APPBP2*, *STAU1*; Supplementary Material, Table S2). Manhattan and QQ plots have been depicted in Figures 1A and 1B.

The minor alleles of *IL6R* rs4537545^*^T (*β* = 0.091; *P*_discovery_ = 8.39 × 10^−85^), *IL1F10*/*IL1RN* rs6734238^*^G (*β* = 0.025; *P*_discovery_ = 1.45 × 10^−7^) and *HLA-DRB1*/*5* rs660895^*^G (*β* = 0.036; *P*_discovery_ = 1.80 × 10^−9^) associated with increased circulating IL-6 levels (Table 1). Two additional genome-wide significant SNPs in the *IL1R* locus, rs11265618 (*β* = 0.047; *P*_discovery_ = 1.21 × 10^−15^) and rs10796927 (*β* = 0.034; *P*_discovery_ = 1.24 × 10^−11^), in low LD (*r*^2^ < 0.25) with the lead SNP rs4537545 were carried forward for replication, and later conditional analysis as they seemed potential candidates as independent signals.

**Table 1:** Novel and replicated loci associated with circulating IL-6 levels at *P* < 5.0 × 10^−8^ in the combined GWAS meta analyses

Chr	Lead SNP (rsID)	BP (Hg19)	Effect/Other allele	EAF	Beta (SE)	*P*_discovery_	*P*_replication_	*P*_combined_	Annotation	Nearest Genes	*P*_het_ (*I*^2^)
*Novel Loci*											
2q14	rs6734238	113 841 030	G/A	0.42	0.025 (0.005)	1.45 × 10^−7^	3.24 × 10^−5^	1.84 × 10^−11^	Intergenic	*IL1F10/IL1RN*	0.03 (32)
6p21	rs660895	32 577 380	G/A	0.19	0.036 (0.006)	1.80 × 10^−9^	3.38 × 10^−2^	1.55 × 10^−10^	Intergenic	*HLA-DRB5/DRB1*	0.08 (26)
*Replicated Known Locus*											
1q21	rs4537545	154 418 879	T/C	0.39	0.091 (0.005)	8.39 × 10^−85^	7.88 × 10^−37^	1.20 × 10^−122^	Intronic	*IL6R*	<0.01 (72)

Index SNPs that reached *P* < 5 × 10^–8^ in the combined analysis from each locus are reported here. Sample sizes: discovery cohorts, *n* = 52 654; replication cohorts, *n* = 14 774; combined, *n* = 67 428. The effect sizes (*β*) in the discovery phase, given for the effect allele. EAF: effect allele frequency; effect sizes and standard error (SE) values are based on natural log transformed IL6 (pg/ml) levels. Phet: combined meta-analysis heterogeneity *P* value; I^2^: heterogeneity measure.

Overall, 12 SNPs spanning over 9 loci at a *P*_discovery_ < 1 × 10^−5^ in the discovery GWAS meta-analyses were selected for the replication stage (Supplementary Material, Table S2). This included the three index SNPs, two additional SNPs from the 1q21 locus (GWS but in low LD, *r*^2^ < 0.25 with index SNP) plus an additional set of seven statistically suggestive SNPs with a *P*-value of 5 × 10^−8^ < *P* < 1 × 10^−5^ in the discovery meta-analyses (either in low LD, *r*^2^ < 0.25 with the index SNP or independent loci). Additionally, 3 SNPs as negative controls and 3 SNPs in LD (*r*^2^ > 0.25) with the Chr.1 index SNP, to control for possible genotyping errors of index SNP across replication cohorts, were also added to the replication list, yielding 18 SNPs for replication stage (Supplementary Material, Table S3).

Three loci including Chr.1q21 *IL6R*, Chr.6p21 *HLA-DRB1*/*5* and Chr.2q14 *ILF10/IL1RN* replicated at *P*_replication_ < 0.05, reaching GWS; 1q21 rs4537545, *P*_combined_ = 1.20 × 10^−122^; 6p21 rs660895, *P*_combined_ = 1.55 × 10^−10^; and 2q14 rs6734238, *P*_combined_ = 1.84 × 10^−11^ in the combined meta-analyses (Table 1 and Supplementary Material, Table S3). Locus zoom plots available in Figures 1C, 1D, and 1E. The two additional signals at Chr.1q21 *IL6R* locus were replicated at *P*_replication_ = 1.7 × 10^−4^ for rs11265618 and *P* = 0.03 for rs10796927, reaching *P*_combined_ = 2.5 × 10^−9^ and *P*_combined_ = 4.1 × 10^−13^, respectively (Supplementary Material, Table S3). The conditional analysis confirmed that rs11265618 and rs10796927 SNPs were not independent from (Supplementary Material, Table S4) but were driven by the index rs4537545 SNP.

**Figure 2:**
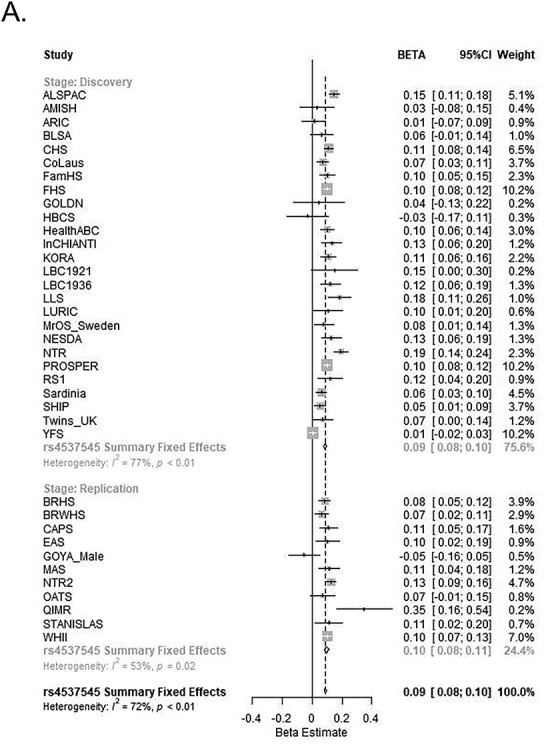

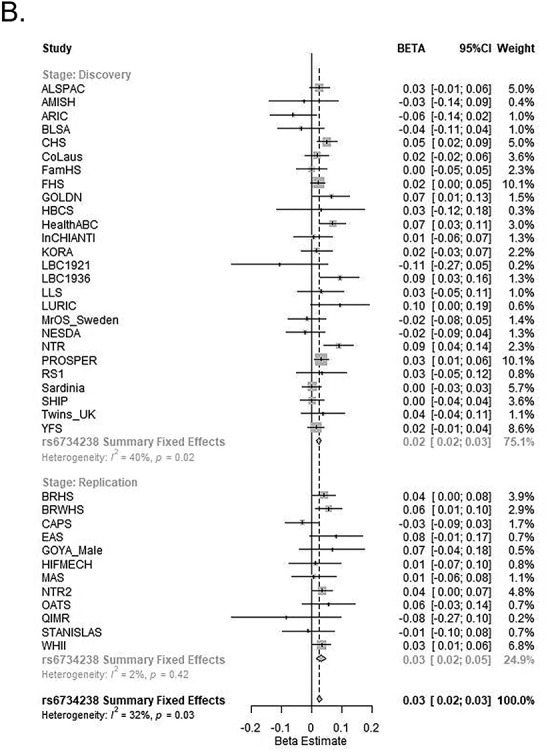

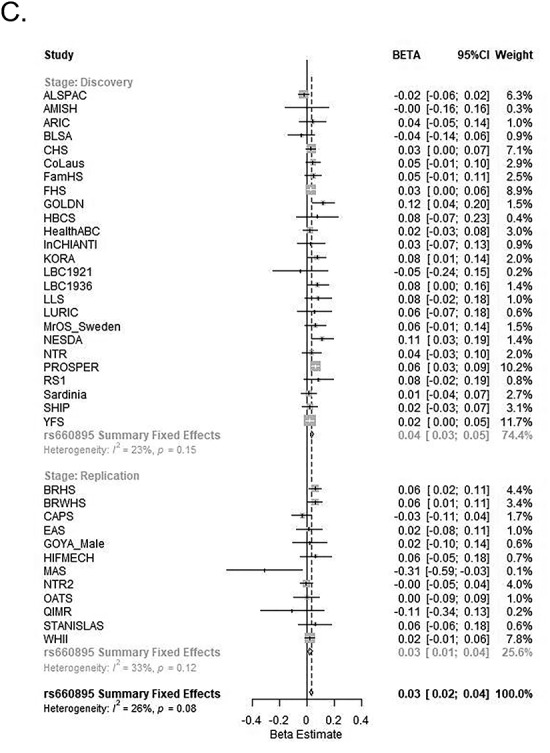
Combined discovery and replication forest plots for the GWAS Index SNPs. Forrest plots for (**A**) *IL6R* rs4537545 (chr. 1q21), (**B**) *IL1RN* rs6734238 (chr. 2q14), (**C**) *HLA-DRB5* rs660895 (chr. 6p21) with discovery, replication and combined effect estimates, 95% CI and weights based on the fixed effects inverse variance meta-analyses.

In both, discovery and replication association analyses, the effect directionality was generally consistent across individual studies for GWS variants, while there was some evidence of borderline heterogeneity in one of the two novel loci (*I*^2^ (*P* value) < 0.05) during the discovery and combined meta-analysis (Table 1, Fig. 2). The imputation quality scores (*r*^2^) for the GWS (index) SNPs for each cohort (discovery and replication) are available in Supplementary Material, Table S5. The other seven SNPs that showed suggestive association in the discovery stage, and expectedly the negative control SNPs did not reach GWS in the combined meta-analyses (Supplementary Material, Table S3).

The three GWAS index SNPs when combined explained approximately 1.06% of the variance in circulating levels of IL-6 using data from the NESDA cohort. The phenotypic variance explained by all the common variants was estimated to be 4.45% using the SumVg method (38).

### Replication of other known/suggestive loci for IL-6

*IL6R* was the only IL6 known locus that we replicate at GWS. *IL1RN* and *HLA-DRB1*, our primary findings have been reported as suggestive loci (1 × 10^−6^ < *P* < 1 × 10^−4^) by Shah *et al.* while some known/suggestive IL6 loci (*ABO*, *BUD13*, *TRIB3* and *SEZ6L*) did not replicate (*P*_discovery_ > 0.05) in the current study.

### SNP functionality

We looked up SNPs in LD with the index SNPs from the immunologically associated loci including *IL-6R*, rs4537545, 1q21; *IL1F10*, *IL1RN*, rs6734238, 2q14, intergenic; and *HLA-DRB1/DRB5*, rs660895, 6p21, intergenic. The search for functional/missense variants in high LD (*r*^2^ > 0.8) with the lead SNPs led to the identification of only one nonsynonymous rs2228145 SNP in LD (*r*^2^ = 0.95) with the rs4537545 index SNP from the *IL6R* locus. We used the Combined Annotation-Dependent Depletion (CADD) database to identify the functionality, i.e. deleterious, disease causal, pathogenicity, of rs2228145 in *IL6R*. CADD is an integrative annotation based on multiple genomic features scored into a single metric (39). *IL6R* missense the rs2228145 variant has a CADD score of 15.98 (https://cadd.gs.washington.edu).

### Associations with other traits and gene expression data

Genome-wide significant associations between IL6-associated top SNPs and other traits, and gene expression, data were mined using the Pheno Scanner v2 database (accessed, October 2020).

GWAS-based *IL1F10*/*IL1RN* rs6734238^*^G allele has been associated with increased levels of serum C-reactive protein (CRP) and decreased fibrinogen levels, and blood cell traits in recent GWAS reports (40,41) (PMID:27863252; Supplementary Material, Table S6).

*HLA-DRB1/DRB5* rs660895^*^G allele is associated with increased risk of rheumatoid arthritis (RA) in Europeans and Asians (42), IgA nephropathy in Asians (43), while the decreased risk of ulcerative colitis and inflammatory bowel disease (IBD) (44). *IL-6R* rs4537545^*^T allele has been associated with increased circulating CRP levels (45), a decreased risk of RA (42) in mixed ancestries, while an increased risk of diabetes and asthma from the UK Biobank Neale’s lab rapid GWAS (See Web Resources; Supplementary Material, Table S6). *IL6R* rs4537545T^*^ allele is also associated with C-reactive protein, allergic disease, rheumatoid arthritis and coronary artery disease (Supplementary Material, Table S6).

#### Gene expression

*IL1F10/IL1RN* rs6734238 is associated with IL1F10/IL1RN expression levels in the skin, peripheral blood and whole blood (*P* < 5.0 × 10^−8^; Supplementary Material, Table S7). *HLA-DRB1/DRB5* rs660895 has been associated with HLA-DRB1/DRB5/DRB6/DQB1/DQB2 expression levels in multiple tissues including peripheral blood, whole blood, monocytes, adipose tissue, thyroid, tibial artery, coronary artery, heart, lung, brain, colon, skeletal muscle, tibial nerve, skin and lymphoblastoid cell lines (*P* < 5.0 × 10^−8^; Supplementary Material, Table S7)**.***IL6R* rs4537545 SNP is also associated with IL6R expression levels in peripheral and whole blood (*P* < 5.0 × 10^−8^; Supplementary Material, Table S7).

### Power estimates

Based on power calculator and assumptions mentioned under methods section, the estimated power for the 2 novel index SNPs was 98.3% rs6734238 (effect allele frequency, EAF: 0.42), and 76.9% rs660895 (EAF: 0.19), respectively.

## Discussion

We performed the largest (to date) GWAS meta-analysis for circulating IL-6 levels, which includes 66 341 individuals of European ancestry. We identified three loci associated with levels of circulating of IL-6 in the general population amongst which two are novel (Chr6p21, and Chr2q14), located in/nearby genes (*HLA-DRB1* and *IL1RN/IL-38*) with inflammatory roles explaining up to 1.06% variance.

The strongest associated SNP, interleukin 6 receptor (*IL-6R)* rs4537545 at the 1q21 locus, is in high LD (*r*^2^ = 0.95) with a missense *IL-6R* SNP rs2228145 (D358A) that results in an amino acid substitution at position 358 (Asp → Ala) on the extracellular domain of IL-6R and a high CADD score suggesting that the variant is pathogenic or functional or deleterious (among top 10% variants of the genome). The missense SNP is known to impair the responsiveness of cells targeted by IL-6 (46) by reducing IL-6R expression on cell surfaces (47), and increasing levels of soluble IL-6R in individuals homozygous for this mutation (48,49). Recently it has been demonstrated that increased levels of sIL-6R induced by this variant can be explained by ectodomain shedding off IL-6R, a mechanism in which membrane-associated proteins are rapidly converted into soluble effectors whereby simultaneously cell surface expression of the same protein is reduced (50). Increased levels of sIL-6R may act as a counter-balance to limit exaggerated IL-6 signaling and may explain the protective effect of the 358A allele for various cardiovascular diseases including coronary artery disease (CAD) (51–53), atrial fibrillation (54), lung function in asthmatics (55) and abdominal aortic aneurysm (56) as well as RA (57). However, in contrast with this finding, the IL-6-sIL-6R complex itself is capable of transducing IL-6 signaling to non-IL-6R expressing cells, known as trans-signaling (58), and it is this mechanism, as opposed to classic signaling, that is linked to chronic inflammatory disorders including IBD and RA (59). Blocking IL-6 signaling cascades can be achieved by using an IL-6R specific inhibitor in the form of a monoclonal antibody, tocilizumab, which is a widely used therapy in the treatment of RA. Several variants in IL-6R, including rs2228145, may assist in the prediction of patient response to tocilizumab in RA (60). The rs4537545^*^T allele that is associated with IL6 levels is known to associate with increased circulating CRP levels (61) and a decreased risk of RA (42) in studies comprising mixed ancestries. Moreover, this SNP has been associated with IL6R expression in peripheral blood, skin, brain and adipose tissue (Supplementary Material, Table S7). The causal involvement of IL-6 levels in disease remains to be elucidated, but a recent study using a Mendelian randomisation (MR) approach did demonstrate that by using this SNP as instrumental variable, modelling the effects of tocilizumab, that IL-6R signalling has a causal effect on CAD (52). On the other hand, pleiotropic nature of the IL-6R locus, influencing IL-6, CRP and fibrinogen levels, prohibits instrumental variable analysis and attribution of causality to one particular intermediate. Finally, several other genes encompass the 1q21 locus, including Src homology 2 domain containing E (*SHE*), and Tudor domain containing 10 (*TDRD10*), but have been ruled out to play a role at this locus (33).

At the identified chromosome 2 locus the lead SNP, rs6734238, is intergenic and has also been associated with circulating CRP and fibrinogen levels (40,41,62). The nearest genes to this locus are the interleukin 1 family member 10 (*IL1F10*, distance = 7.6 kb, currently known as *IL-38*) and interleukin 1 receptor antagonist (*IL1RN*, distance = 34.4 kb). *IL1F10/IL-38* and *IL1RN* variants (rs6759676 and rs4251961) in partial LD with the lead SNP (*r*^2^_LD_:0.10 and 0.61) have been recently reported to be protective against the development of insulin resistance (63). This further supports the molecular mechanisms behind IL-6-mediated insulin secretion via glucagon-like peptide 1 (GLP-1) (64) contributing to type 2 diabetes (T2D) pathophysiology. For IL-6 specifically, it has been found that synthesis increases when dendritic cells are stimulated by bacterial lipopolysaccharides (LPS) in the presence of *IL1F10* (65). *IL-1RN* is another member of the interleukin 1 cytokine family, with suggestive evidence for involvement in determining IL-6 levels in the blood. One study found significant associations of *IL-1RN* rs4251961 with plasma CRP and IL-6 levels, albeit not independently replicated and not genome-wide significant (*P* = 1 × 10^−4^ and *P* = 0.004) (66). Our lead SNP was not in high LD (*r*^2^ < 0.8) with variants in either neighboring genes and therefore in conjunction with its intergenic position, identifying a causal variant in this locus remains non-trivial.

The 6p21 rs660895, which was identified, resides within the HLA region, which forms one of the most complex genomic regions to study due to its large LD blocks and sequence diversity. This region has some population substructure in Europeans, which may have influenced the results; however, (1) each cohort population substructure adjustment was applied, followed by genomic correction for overall discovery stage meta-analyses. Thus, we reduced the chances that the population substructure may have had on this locus. The nearest genes to the index SNP, *HLA-DRB1* (distance = 19.8 kb) and *HLA-DQA1* (distance = 27.8 kb) are both histocompatibility complex genes encoding proteins that form cell surface complexes for certain immune system cells helping in antigen presentation to trigger an immune response. It is noteworthy that variations at this locus code for antigen-presenting complexes (APCs), which have been previously associated with diseases having a dysfunctional immune system; while we report for the first time that there exists also a strong association of this locus with circulating cytokine levels. Therefore, the association of this locus with the disease may corroborate through its effect through IL6 levels. One high-LD SNP (rs9272422, *r*^2^ = 0.82 with our index SNP, rs660895) residing in the promoter region of *HLA-DQA1* support this hypothesis and has been identified previously for systemic lupus erythematosus (67) and ulcerative colitis (68). rs660895^*^G allele is associated with increased risk of RA in Europeans and Asians (42), IgA nephropathy in Asians (43), while the decreased risk of ulcerative colitis and IBD. (44)

Various studies aimed to identify genetic variation underlying levels of IL-6 (22–26) have found genome-wide significant associations in the *IL-6R* and *ABO* genes. The study performed by Shah and colleagues (33) found suggestive evidence (non-GWS; *P* = 3.8 × 10^−6^, respectively) for additional loci, including *ABO*, *BUD13*, *TRIB3* and *SEZ6L*, none of which replicate in the current study (*P*_discovery_ > 0.05) indicating that these might be false-positive findings due to low sample size (~7800) or loci with sex-specific effects (associations based on women dominant population) or due to technical shortcomings with measurement assay (*ABO* locus).

It is surprising that even with increased statistical power (*n*_discovery_ = 52 654; *n*_replication_ = 14 774) in the current study (compared to the previous IL6 GWAS) (33), we could identify three genetic loci (1q21, 2q14 and 6p21) accounting for ~1% of the genetic variance for circulating IL-6 levels. According to the current estimates, the heritability levels for IL6 levels range between 15 and 61%, suggesting that an enormous increase in sample sizes would be required to identify additional variants explaining this remaining heritability. Multiple explanations for this so-called missing heritability phenomenon have been proposed in the past, which can be sought in rare or low frequency coding variants as observed for a similar metabolic quantitative trait by us (69) or can be explained by non-additive effects, which may cause inflated estimates of heritability. Plausible evidence for other sources of unexplained heritability that have been found are epigenetic changes, and haplotypes of common SNPs.

Collectively, our results provided additional insights into the biology of circulating IL-6. We identify new loci, limited by common variants in the Hap Map Reference panel. Albeit this is comparable to the 1000 genomes reference panel (70) but narrower compared to some newly available panels that show greater variant coverage in numbers and frequency range. Future studies are recommended to aim for identification of additional common but also rare variants, by firstly using richer imputation panels, such as UK10K project or the Haplotype Reference Consortium, a strategy that holds great promise, and secondly by making use of genetically isolated populations. Thirdly, we would like to stress the importance of phenotype harmonization. As we identified genome-wide variants in the *ABO* locus, in four studies participating in the discovery, but not in the remaining 22 cohorts, there is a strong indication that this locus may be assay specific. However, a proper demonstration of this hypothesis would require further testing, including repeating the GWAS in *ABO*-positive cohorts using a different IL-6 assay. Indeed it is emerging that the *ABO* locus has pleiotropic effects on many different traits and diseases (71), which would suggest a more thorough analysis before disregarding this signal. Also, conventionally increasing sample sizes without correction for population substructures may raise heterogeneity within populations (72), likely concealing the SNPs that affect particular subgroups. Future specific studies should counter the widely held assumption of unconditional risk alleles of complex traits and focus on the importance of studying more homogenous subgroups to, for example, investigate the age-dependent effect of genetic variants (73,74). Here, while further exploring the pleiotropic effect of IL-6-related variants, we identified phenotypes differentially regulated by diverse variants in the 1q21 locus. Biologic systems are dynamic complex networks and are evolving through lifespan and investigating the interrelationships existing between phenotypes as well as between genetic variations and phenotypic variations has the potential for uncovering the complex mechanisms. This is the case here for IL-6 and tailored methodologies should be devoted to the study of such traits, hopefully resulting in clinically significant breakthroughs. Future collaborative efforts therefore should strive to use well-calibrated assays, *z*-standardized protocols for sample handling, and processing (75), though this will be difficult to achieve in practice. Lastly, we have attempted to perform formal association-based causal analysis to identify the likely causal loci, using the DEPICT approach; unfortunately, instead with only 2 novel GWS findings, our analyses were underpowered and thus not included. We also mine the gene expression and eQTL data for the identified SNPs using established databases; however, we were unable control for random co-localization signals or other confounders as we had limited access to summary level data. In conclusion, we identify two novel common genetic variants associated with circulating IL-6 levels that may influence the pathophysiology of complex cardio-metabolic, psychiatric and immunological traits, among individuals of European ancestry. This is a step further towards unravelling new biological pathways and potential therapeutic targets that can be developed for the IL-6-related disorders, while suggesting looking deeper into the genome for coding variants (rare and common) having larger individual effects (Figs 1 and 2).

## Material and Methods

### Discovery stage

#### Study populations

The overall study design (Supplementary Material, Fig. S1) involved the discovery cohorts with 53 893 individuals. After overlapping individuals with available genotype and phenotype data, the discovery stage included 52 654 individuals from 26 cohorts of European ancestry listed under Supplementary Material, Table S8, described in Supplementary Text S1 and study summary characteristics in Supplementary Material, Table S9. Only population-based samples or healthy controls from case–control studies were included in the final analyses.

#### Serum IL-6 measurements

Each study typically collected venous blood samples stored below −80°C until the time of measurement using various types of immunoassays and expressed as pg/ml as presented in Supplementary Material, Table S10. The trait transformation and phenotype data quality control (QC) were presented by Supplementary Material, Text S3 (Supplementary Material, Text S3.1 and S3.2). In brief, participating cohorts have checked for the percentage of missingness in IL6 measurements and evaluated for indices of QC (Supplementary Material, Text S3.2), yielded the final number of participants with available validated IL6 levels, of whom those with available genotype data were included in the study as characterized in Supplementary Material, Table S9 and in Supplementary Material, Text S1 and S2.

#### Genotyping and imputation

Each participating cohort performed genome-wide genotyping using a variety of genotyping platforms and applied a predefined quality control (QC) of genotype data (Supplementary Material, Table S11) followed by performing imputation of non-genotyped genetic variants, on the backbone of haplotypes inferred from the Hapmap Phase II reference panel (NCBI Build 36), and using statistical software such as IMPUTE (75), MACH, Minimac (76) or BIMBAM (77) (Supplementary Material, Table S11). Each cohort was recommended a set of general SNP quality filters including MAF < 0.01; Hardy Wienberg Equilibrium (HWE) *P* ≤ 10^−6^; imputation quality *r*^2^ ≤ 0.3; and genotyping call rate <0.95 (Supplementary Material, Fig. S1). Once we received summary results from each participating study, we ran a series of QC checks. Firstly, these included a set standard checks, including the imputation quality filters (basis the imputation program used and/or *r*^2^_imputation_ < 0.3 were excluded), and then checks for genomic inflation (quantile–quantile or QQ plots). We adapted filter thresholds per cohort to reduce any observed deviation from the null while missing SNP loss due to the QC process. Finally, ~2.45 million (2 454 025) common SNPs were part of the discovery meta-analysis.

### Statistical methods

#### GWAS analysis

Each study conducted an independent GWAS analysis between SNPs and natural log-transformed values of serum IL-6 levels following a predefined analysis plan (Supplementary Material, Methods S4). Association analyses were conducted using linear regression model, or linear mixed effect models to account for familial correlation when warranted, with additive genetic effects, accounting for imputation uncertainty while adjusting for age, sex, population substructure (through study-specific principal components) and/or study-specific site, when necessary. GWAS summary result obtained from each cohort underwent a series of QC checks using the QCGWAS package in R (78) (Supplementary Material, Text S3 and Supplementary Material, Fig. S1). Being aware of the potential false-positive association in the *ABO* region on chromosome 9 (28,30), while using an R&D systems high-sensitivity assay kit to measure IL6 levels (R&D systems, Minneapolis, MN, USA), four (out of 22) discovery cohorts that observed genome-wide significant results in the *ABO* locus were asked to rerun the GWAS analysis conditional on the top *ABO* SNP (i.e. rs8176704) before including them in the final discovery meta-analysis (Supplementary Material, Text S3.3).

#### Discovery GWAS meta-analyses

Individual GWAS results from 26 European studies were meta-analyzed using the inverse variance weighted, fixed-effects method as implemented in GWAMA while applying the double genomic control (GC) correction for population stratification, i.e. first to each study individually and subsequently also to the pooled results after meta-analysis (79).

Regional association plots for the discovered loci were generated through the LocusZoom (78) tool. We used the SNAP tool (80) to perform the pairwise LD checks (HapMap release 22 data) and to verify low LD with secondary signals. All SNPs selected for the replication stage had to fulfill the following criteria: (1) having an association *P*_discovery_ ≤ 1 × 10^−5^ and being in very low LD with the index SNP (*r*^2^ < 0.2) and (2) available in at least 50% of study cohorts.

### Replication and combined meta-analysis

#### Study population, phenotyping and QC

The overall study (Supplementary Material, Fig. S1) comprised 15 785 individuals for replication. After removing individuals with missing data, the replication analyses were performed using a combination of *in silico* and *de novo* genotyping in 14 774 individuals from 12 cohorts of European ancestry as described in Supplementary Material, Text S2. Similar QC (Supplementary Material, Text S3 and Supplementary Material, Table S11) and statistical checks were made as in the discovery stage.

Venous blood samples (serum or plasma) were collected and stored at −80°C. Serum/plasma IL-6 levels (pg/ml) were measured using various immunoassay methods described in Supplementary Material, Table S10. Each cohort tested the selected SNPs using the same statistical model as for the discovery association analyses (Supplementary Material, Fig. S1). Effect size estimates of all replication SNPs from each replication study were compared with the effect size estimates from the discovery meta-analyses. When effect sizes from individual cohorts did not align, we excluded these cohorts from the replication meta-analyses (*n*_cohorts_ = 3). To account for the inter-study assay differences insensitivity, we combined results across the replication studies using a fixed effect sample size weighted Z-score meta-analysis as implemented in the METAL package (https://genome.sph.umich.edu/wiki/METAL) (81).

The summary GWAS meta-analyses result from the discovery and replication stages were then used to perform a combined (discovery + replication) GWAS meta-analysis using a sample size weighted *Z*-score method. Test for heterogeneity was also performed as part of the meta-analysis package using METAL where *I*^2^ statistic denoting the percentage of variation across studies was estimated (*I*^2^ = 100% × (*Q −* df)/*Q*) where *Q* is the Chi-Square statistic. Significance for heterogeneity was denoted by the heterogeneity (or Het*_P_*) *P* values. Variants that were significant in the replication meta-analysis at *P* < 0.05 and had an overall *P*_combined_ < 5 × 10^−8^ in the combined meta-analysis were considered statistically GWS. SNPs within the range of 1 Mb (or 10^6^ bases) on either side of the most significant (i.e. index) SNP (with LD, *r*^2^ > 0.25) were considered part of the same locus, whereas those in low LD (*r*^2^ < 0.25) were tested if they were independent using conditional analysis.

#### Conditional analysis

We performed an approximate joint conditional analysis to identify distinct signals in a specific chromosomal region as implemented in GCTA (82) using high-quality genome-wide genotyped/imputed reference data from two studies (NEtherlands Study of Depression and Anxiety (NESDA) from the Netherlands and/or Genetics of Obesity in Young Adults (GOYA) from Denmark) to estimate linkage disequilibrium (LD) (83) between SNPs.

Conditional analysis for identification of independent signals was performed on GWS SNPs ( ±1 Mb to the index SNP and having low LD, *r*^2^ < 0.2 with the index SNP) using summary statistics from the discovery GWAS meta-analysis data (—COJO option in GCTA) after confirming the GWS loci from the combined meta-analysis.

#### Heritability estimates

We approximated the variance explained by all distinct lead SNPs from the meta-analysis using the following formula:

$$\sum_{i=1}^n\frac{\beta_i^2 \cdot 2\cdot{\mathrm{EAF}}_i\cdot \left(1-{\mathrm{EAF}}_i\right)}{\sigma^2\left(\mathrm{residuals}\left(\ln \left(\mathrm{IL}6\right)\right)\right)}$$

where EAF is the effect allele frequency, ${\beta}_i$ the effect size of the individual variants, and *n* is the total number of lead variants. The current formula may overestimate the variance to a small extent as some level of SNP correlation was existent (LD *r*^2^ < 0.25). The variance of the residuals of ln (IL-6) was calculated using data from the NESDA cohort (*n* = 2517). The total common SNP heritability of serum IL-6 levels explained by all GWAS variants was estimated using the observed *Z*-statistics from the discovery analyses for a subset of pruned SNPs. Following the original method (SumVg) (38), we pruned the imputed (based on the 1000G *Phase1 Integrated Release, Version 3, 2012.04.30* reference panel) genotypes of the NESDA cohort using PLINK v1.07 (84), by removing correlated SNPs at *r*^2^ > 0.25 within a 100-SNP sliding window, and with a step size of 25 SNPs per forwarding slide. This resulted in a pruned set of 163 459 SNPs.

#### SNP mapping and functionality

We searched for variants in high LD (*r*^2^ > 0.8) within a 1 Mb region on either side of the lead SNPs using 1000 Genomes sequence data (Phase1 Integrated Release, Version 3, 2012.04.30), utilizing tools available in Liftover (85), VCFtools (86) and clumping in PLINK (84). We subsequently annotated these variants using ANNOVAR (87) with the RefSeq (88) database for variant function and genic residence or distance. We used the Combined Annotation-Dependent Depletion (CADD) database to identify the functionality, i.e. deleterious, disease causal, pathogenicity, for the index SNPs.

#### Associations with other traits and gene expression data

PhenoScanner v2 (89) data mining tool was used (Access date October 2020) to identify existing GWS (at *P* < 5 × 10^−8^) associations between IL6 identified SNPs and other traits, and gene expression/eQTLs data.

### Power calculation

We used GWAs power estimator (see Web Resources) by assuming a relative risk of 1.10 (or an effect estimate of 0.10), given *N* = 66 000, alpha (*P*-value) = 5 × 10^−8^ (also GCTA power calculator, Supplementary Text S3.5).

## Supplementary Material

IL6_GWAS_Supp_Tables_HMG_11012021_ddab023

Supplementary_Material_IL6_GWAS_11012021_ddab023

## Web Resources

QCGWAS, https://cran.r-project.org/web/packages/QCGWAS/index.html

GWAMA, http://www.well.ox.ac.uk/gwama/

METAL, http://csg.sph.umich.edu//abecasis/metal/

GCTA, http://www.complextraitgenomics.com/software/gcta/

LocusZoom, http://csg.sph.umich.edu/locuszoom/

1000 Genomes, ftp://ftp.1000genomes.ebi.ac.uk/vol1/ftp/release/20110521/

PLINK, http://pngu.mgh.harvard.edu/purcell/plink/

VCFtools, http://vcftools.sourceforge.net/

ANNOVAR, http://www.openbioinformatics.org/annovar/

PhenoScanner, http://www.phenoscanner.medschl.cam.ac.uk/phenoscanner

Power calculations: http://csg.sph.umich.edu/abecasis/cats/gas_power_calculator/index.html

The UK Biobank Neale’s lab rapid GWAS: (http://www.nealelab.is/blog/2017/7/19/rapid-gwas-of-thousands-of-phenotypes-for-337000-samples-in-the-uk-biobank).

## References

[1] Kishimoto, T. (2010) IL-6: from its discovery to clinical applications. Int. Immunol., 22, 347–352. 20410258 10.1093/intimm/dxq030

[2] Nishimoto, N. and Kishimoto, T. (2006) Interleukin 6: from bench to bedside. Nat. Clin. Pract. Rheumatol., 2, 619–626. 17075601 10.1038/ncprheum0338

[3] Brocker, C., Thompson, D., Matsumoto, A., Nebert, D.W. and Vasiliou, V. (2010) Evolutionary divergence and functions of the human interleukin (IL) gene family. Hum. Genomics, 5, 30–55. 21106488 10.1186/1479-7364-5-1-30 PMC3390169

[4] Gelinas, L., Falkenham, A., Oxner, A., Sopel, M. and Legare, J.F. (2011) Highly purified human peripheral blood monocytes produce IL-6 but not TNFalpha in response to angiotensin II. J. Renin-Angiotensin-Aldosterone Syst., 12, 295–303. 21393356 10.1177/1470320310391332

[5] Kitani, A., Hara, M., Hirose, T., Harigai, M., Suzuki, K., Kawakami, M., Kawaguchi, Y., Hidaka, T., Kawagoe, M. and Nakamura, H. (1992) Autostimulatory effects of IL-6 on excessive B cell differentiation in patients with systemic lupus erythematosus: analysis of IL-6 production and IL-6R expression. Clin. Exp. Immunol., 88, 75–83. 1563109 10.1111/j.1365-2249.1992.tb03042.x PMC1554365

[6] Li, T. and He, S. (2006) Induction of IL-6 release from human T cells by PAR-1 and PAR-2 agonists. Immunol. Cell Biol., 84, 461–466. 16869943 10.1111/j.1440-1711.2006.01456.x

[7] Ng, E.K., Panesar, N., Longo, W.E., Shapiro, M.J., Kaminski, D.L., Tolman, K.C. and Mazuski, J.E. (2003) Human intestinal epithelial and smooth muscle cells are potent producers of IL-6. Mediat. Inflamm., 12, 3–8. 10.1080/0962935031000096917 PMC1781593 12745542

[8] Fain, J.N. (2006) Release of interleukins and other inflammatory cytokines by human adipose tissue is enhanced in obesity and primarily due to the nonfat cells. Vitam. Horm., 74, 443–477. 17027526 10.1016/S0083-6729(06)74018-3

[9] Podor, T.J., Jirik, F.R., Loskutoff, D.J., Carson, D.A. and Lotz, M. (1989) Human endothelial cells produce IL-6. Lack of responses to exogenous IL-6. Ann. N. Y. Acad. Sci., 557, 374–385 discussion 386-377. 2660697

[10] Sanchez, C., Gabay, O., Salvat, C., Henrotin, Y.E. and Berenbaum, F. (2009) Mechanical loading highly increases IL-6 production and decreases OPG expression by osteoblasts. Osteoarthr. Cartil., 17, 473–481. 10.1016/j.joca.2008.09.007 18974013

[11] Haddy, N., Sass, C., Maumus, S., Marie, B., Droesch, S., Siest, G., Lambert, D. and Visvikis, S. (2005) Biological variations, genetic polymorphisms and familial resemblance of TNF-alpha and IL-6 concentrations: STANISLAS cohort. Eur. J. Hum. Genet., 13, 109–117. 15523500 10.1038/sj.ejhg.5201294

[12] Madhok, R., Crilly, A., Watson, J. and Capell, H.A. (1993) Serum interleukin 6 levels in rheumatoid arthritis: correlations with clinical and laboratory indices of disease activity. Ann. Rheum. Dis., 52, 232–234. 8484679 10.1136/ard.52.3.232 PMC1005024

[13] de Benedetti, F., Massa, M., Robbioni, P., Ravelli, A., Burgio, G.R. and Martini, A. (1991) Correlation of serum interleukin-6 levels with joint involvement and thrombocytosis in systemic juvenile rheumatoid arthritis. Arthritis Rheum., 34, 1158–1163. 1930333 10.1002/art.1780340912

[14] Ahluwalia, T.S., Kilpelainen, T.O., Singh, S. and Rossing, P. (2019) Editorial: novel biomarkers for type 2 diabetes. Front Endocrinol (Lausanne), 10, 649. 31611845 10.3389/fendo.2019.00649 PMC6776920

[15] Cesari, M., Penninx, B.W., Newman, A.B., Kritchevsky, S.B., Nicklas, B.J., Sutton-Tyrrell, K., Rubin, S.M., Ding, J., Simonsick, E.M., Harris, T.B. and Pahor, M. (2003) Inflammatory markers and onset of cardiovascular events: results from the health ABC study. Circulation, 108, 2317–2322. 14568895 10.1161/01.CIR.0000097109.90783.FC

[16] Qu, D., Liu, J., Lau, C.W. and Huang, Y. (2014) IL-6 in diabetes and cardiovascular complications. Br. J. Pharmacol., 171, 3595–3603. 24697653 10.1111/bph.12713 PMC4128059

[17] Mauer, J., Denson, J.L. and Bruning, J.C. (2015) Versatile functions for IL-6 in metabolism and cancer. Trends Immunol., 36, 92–101. 25616716 10.1016/j.it.2014.12.008

[18] Mucha, S., Baurecht, H., Novak, N., Rodríguez, E., Bej, S., Mayr, G., Emmert, H., Stölzl, D., Gerdes, S., Jung, E.S. et al. (2020) Protein-coding variants contribute to the risk of atopic dermatitis and skin-specific gene expression. J. Allergy Clin. Immunol., 145, 1208–1218. 31707051 10.1016/j.jaci.2019.10.030

[19] Zhang, C., Wu, Z., Zhao, G., Wang, F. and Fang, Y. (2016) Identification of IL6 as a susceptibility gene for major depressive disorder. Sci. Rep., 6, 31264. 27502736 10.1038/srep31264 PMC4977523

[20] Calabrese, L.H. and Rose-John, S. (2014) IL-6 biology: implications for clinical targeting in rheumatic disease. Nat. Rev. Rheumatol., 10, 720–727. 25136784 10.1038/nrrheum.2014.127

[21] Scheinecker, C., Smolen, J., Yasothan, U., Stoll, J. and Kirkpatrick, P. (2009) Tocilizumab. Nat. Rev. Drug Discov., 8, 273–274. 19337270 10.1038/nrd2863

[22] Revez, J.A., Bain, L.M., Watson, R.M., Towers, M., Collins, T., Killian, K.J., O'Byrne, P.M., Gauvreau, G.M., Upham, J.W. and Ferreira, M.A. (2019) Effects of interleukin-6 receptor blockade on allergen-induced airway responses in mild asthmatics. Clin. Transl. Immunol., 8, e1044. 10.1002/cti2.1044 PMC6566140 31223480

[23] Yazici, Y., Curtis, J.R., Ince, A., Baraf, H., Malamet, R.L., Teng, L.L. and Kavanaugh, A. (2012) Efficacy of tocilizumab in patients with moderate to severe active rheumatoid arthritis and a previous inadequate response to disease-modifying antirheumatic drugs: the ROSE study. Ann. Rheum. Dis., 71, 198–205. 21949007 10.1136/ard.2010.148700

[24] Yokota, S., Imagawa, T., Mori, M., Miyamae, T., Aihara, Y., Takei, S., Iwata, N., Umebayashi, H., Murata, T., Miyoshi, M. et al. (2008) Efficacy and safety of tocilizumab in patients with systemic-onset juvenile idiopathic arthritis: a randomised, double-blind, placebo-controlled, withdrawal phase III trial. Lancet, 371, 998–1006. 18358927 10.1016/S0140-6736(08)60454-7

[25] Gupta, S., Wang, W., Hayek, S.S., Chan, L., Mathews, K.S., Melamed, M.L., Brenner, S.K., Leonberg-Yoo, A., Schenck, E.J., Radbel, J. et al. (2021) Association between early treatment with tocilizumab and mortality among critically ill patients with COVID-19. JAMA Intern. Med., 181, 41–51. 10.1001/jamainternmed.2020.6252 PMC7577201 33080002

[26] Worns, M.A., Victor, A., Galle, P.R. and Hohler, T. (2006) Genetic and environmental contributions to plasma C-reactive protein and interleukin-6 levels--a study in twins. Genes Immun., 7, 600–605. 16900203 10.1038/sj.gene.6364330

[27] Sas, A.A., Jamshidi, Y., Zheng, D., Wu, T., Korf, J., Alizadeh, B.Z., Spector, T.D. and Snieder, H. (2012) The age-dependency of genetic and environmental influences on serum cytokine levels: a twin study. Cytokine, 60, 108–113. 22673037 10.1016/j.cyto.2012.04.047

[28] Neijts, M., van Dongen, J., Kluft, C., Boomsma, D.I., Willemsen, G. and de Geus, E.J. (2013) Genetic architecture of the pro-inflammatory state in an extended twin-family design. Twin Res. Hum. Genet., 16, 931–940. 23953347 10.1017/thg.2013.58

[29] Amaral, W.Z., Krueger, R.F., Ryff, C.D. and Coe, C.L. (2015) Genetic and environmental determinants of population variation in interleukin-6, its soluble receptor and C-reactive protein: insights from identical and fraternal twins. Brain Behav. Immun., 49, 171–181. 26086344 10.1016/j.bbi.2015.05.010 PMC4567498

[30] Melzer, D., Perry, J.R., Hernandez, D., Corsi, A.M., Stevens, K., Rafferty, I., Lauretani, F., Murray, A., Gibbs, J.R., Paolisso, G. et al. (2008) A genome-wide association study identifies protein quantitative trait loci (pQTLs). PLoS Genet., 4, e1000072. 18464913 10.1371/journal.pgen.1000072 PMC2362067

[31] Naitza, S., Porcu, E., Steri, M., Taub, D.D., Mulas, A., Xiao, X., Strait, J., Dei, M., Lai, S., Busonero, F. et al. (2012) A genome-wide association scan on the levels of markers of inflammation in Sardinians reveals associations that underpin its complex regulation. PLoS Genet., 8, e1002480. 22291609 10.1371/journal.pgen.1002480 PMC3266885

[32] Comuzzie, A.G., Cole, S.A., Laston, S.L., Voruganti, V.S., Haack, K., Gibbs, R.A. and Butte, N.F. (2012) Novel genetic loci identified for the pathophysiology of childhood obesity in the Hispanic population. PLoS One, 7, e51954. 23251661 10.1371/journal.pone.0051954 PMC3522587

[33] Shah, T., Zabaneh, D., Gaunt, T., Swerdlow, D.I., Shah, S., Talmud, P.J., Day, I.N., Whittaker, J., Holmes, M.V., Sofat, R. et al. (2013) Gene-centric analysis identifies variants associated with interleukin-6 levels and shared pathways with other inflammation markers. Circ. Cardiovasc. Genet., 6, 163–170. 23505291 10.1161/CIRCGENETICS.112.964254

[34] Sidore, C., Busonero, F., Maschio, A., Porcu, E., Naitza, S., Zoledziewska, M., Mulas, A., Pistis, G., Steri, M., Danjou, F. et al. (2015) Genome sequencing elucidates Sardinian genetic architecture and augments association analyses for lipid and blood inflammatory markers. Nat. Genet., 47, 1272–1281. 26366554 10.1038/ng.3368 PMC4627508

[35] Denny, J.C., Ritchie, M.D., Basford, M.A., Pulley, J.M., Bastarache, L., Brown-Gentry, K., Wang, D., Masys, D.R., Roden, D.M. and Crawford, D.C. (2010) PheWAS: demonstrating the feasibility of a phenome-wide scan to discover gene-disease associations. Bioinformatics, 26, 1205–1210. 20335276 10.1093/bioinformatics/btq126 PMC2859132

[36] Pendergrass, S.A., Brown-Gentry, K., Dudek, S.M., Torstenson, E.S., Ambite, J.L., Avery, C.L., Buyske, S., Cai, C., Fesinmeyer, M.D., Haiman, C. et al. (2011) The use of phenome-wide association studies (Phe WAS) for exploration of novel genotype-phenotype relationships and pleiotropy discovery. Genet. Epidemiol., 35, 410–422. 21594894 10.1002/gepi.20589 PMC3116446

[37] Pendergrass, S.A., Brown-Gentry, K., Dudek, S., Frase, A., Torstenson, E.S., Goodloe, R., Ambite, J.L., Avery, C.L., Buyske, S., Bůžková, P. et al. (2013) Phenome-wide association study (Phe WAS) for detection of pleiotropy within the population architecture using genomics and epidemiology (PAGE) network. PLoS Genet., 9, e1003087. 23382687 10.1371/journal.pgen.1003087 PMC3561060

[38] So, H.C., Li, M. and Sham, P.C. (2011) Uncovering the total heritability explained by all true susceptibility variants in a genome-wide association study. Genet. Epidemiol., 35, 447–456. 21618601 10.1002/gepi.20593

[39] Rentzsch, P., Witten, D., Cooper, G.M., Shendure, J. and Kircher, M. (2019) CADD: predicting the deleteriousness of variants throughout the human genome. Nucleic Acids Res., 47, D886–D894. 30371827 10.1093/nar/gky1016 PMC6323892

[40] Sabater-Lleal, M., Huang, J., Chasman, D., Naitza, S., Dehghan, A., Johnson, A.D., Teumer, A., Reiner, A.P., Folkersen, L., Basu, S. et al. (2013) Multiethnic meta-analysis of genome-wide association studies in >100 000 subjects identifies 23 fibrinogen-associated loci but no strong evidence of a causal association between circulating fibrinogen and cardiovascular disease. Circulation, 128, 1310–1324. 23969696 10.1161/CIRCULATIONAHA.113.002251 PMC3842025

[41] Dehghan, A., Dupuis, J., Barbalic, M., Bis, J.C., Eiriksdottir, G., Lu, C., Pellikka, N., Wallaschofski, H., Kettunen, J., Henneman, P. et al. (2011) Meta-analysis of genome-wide association studies in >80 000 subjects identifies multiple loci for C-reactive protein levels. Circulation, 123, 731–738. 21300955 10.1161/CIRCULATIONAHA.110.948570 PMC3147232

[42] Okada, Y., Wu, D., Trynka, G., Raj, T., Terao, C., Ikari, K., Kochi, Y., Ohmura, K., Suzuki, A., Yoshida, S. et al. (2014) Genetics of rheumatoid arthritis contributes to biology and drug discovery. Nature, 506, 376–381. 24390342 10.1038/nature12873 PMC3944098

[43] Yu, X.Q., Li, M., Zhang, H., Low, H.Q., Wei, X., Wang, J.Q., Sun, L.D., Sim, K.S., Li, Y., Foo, J.N. et al. (2011) A genome-wide association study in Han Chinese identifies multiple susceptibility loci for IgA nephropathy. Nat. Genet., 44, 178–182. 22197929 10.1038/ng.1047

[44] Liu, J.Z., van Sommeren, S., Huang, H., Ng, S.C., Alberts, R., Takahashi, A., Ripke, S., Lee, J.C., Jostins, L., Shah, T. et al. (2015) Association analyses identify 38 susceptibility loci for inflammatory bowel disease and highlight shared genetic risk across populations. Nat. Genet., 47, 979–986. 26192919 10.1038/ng.3359 PMC4881818

[45] Elliott, P., Chambers, J.C., Zhang, W., Clarke, R., Hopewell, J.C., Peden, J.F., Erdmann, J., Braund, P., Engert, J.C., Bennett, D. et al. (2009) Genetic loci associated with C-reactive protein levels and risk of coronary heart disease. JAMA, 302, 37–48. 19567438 10.1001/jama.2009.954 PMC2803020

[46] Ferreira, R.C., Freitag, D.F., Cutler, A.J., Howson, J.M., Rainbow, D.B., Smyth, D.J., Kaptoge, S., Clarke, P., Boreham, C., Coulson, R.M. et al. (2013) Functional IL6R 358Ala allele impairs classical IL-6 receptor signaling and influences risk of diverse inflammatory diseases. PLoS Genet., 9, e1003444. 23593036 10.1371/journal.pgen.1003444 PMC3617094

[47] Stone, K., Woods, E., Szmania, S.M., Stephens, O.W., Garg, T.K., Barlogie, B., Shaughnessy, J.D., Jr., Hall, B., Reddy, M., Hoering, A. et al. (2013) Interleukin-6 receptor polymorphism is prevalent in HIV-negative Castleman disease and is associated with increased soluble interleukin-6 receptor levels. PLoS One, 8, e54610. 23372742 10.1371/journal.pone.0054610 PMC3553080

[48] Rafiq, S., Frayling, T.M., Murray, A., Hurst, A., Stevens, K., Weedon, M.N., Henley, W., Ferrucci, L., Bandinelli, S., Corsi, A.M. et al. (2007) A common variant of the interleukin 6 receptor (IL-6r) gene increases IL-6r and IL-6 levels, without other inflammatory effects. Genes Immun., 8, 552–559. 17671508 10.1038/sj.gene.6364414 PMC2668154

[49] Galicia, J.C., Tai, H., Komatsu, Y., Shimada, Y., Akazawa, K. and Yoshie, H. (2004) Polymorphisms in the IL-6 receptor (IL-6R) gene: strong evidence that serum levels of soluble IL-6R are genetically influenced. Genes Immun., 5, 513–516. 15306846 10.1038/sj.gene.6364120

[50] Hayashida, K., Bartlett, A.H., Chen, Y. and Park, P.W. (2010) Molecular and cellular mechanisms of ectodomain shedding. Anat Rec (Hoboken), 293, 925–937. 20503387 10.1002/ar.20757 PMC4621804

[51] Collaboration, I.R.G.C.E.R.F, Sarwar, N., Butterworth, A.S., Freitag, D.F., Gregson, J., Willeit, P., Gorman, D.N., Gao, P., Saleheen, D., Rendon, A. et al. (2012) Interleukin-6 receptor pathways in coronary heart disease: a collaborative meta-analysis of 82 studies. Lancet, 379, 1205–1213. 22421339 10.1016/S0140-6736(11)61931-4 PMC3316940

[52] Interleukin-6 Receptor Mendelian Randomisation Analysis, C, Swerdlow, D.I., Holmes, M.V., Kuchenbaecker, K.B., Engmann, J.E., Shah, T., Sofat, R., Guo, Y., Chung, C., Peasey, A. et al. (2012) The interleukin-6 receptor as a target for prevention of coronary heart disease: a mendelian randomisation analysis. Lancet, 379, 1214–1224. 22421340 10.1016/S0140-6736(12)60110-X PMC3316968

[53] Consortium, C.A.D., Deloukas, P., Kanoni, S., Willenborg, C., Farrall, M., Assimes, T.L., Thompson, J.R., Ingelsson, E., Saleheen, D., Erdmann, J. et al. (2013) Large-scale association analysis identifies new risk loci for coronary artery disease. Nat. Genet., 45, 25–33. 23202125 10.1038/ng.2480 PMC3679547

[54] Schnabel, R.B., Kerr, K.F., Lubitz, S.A., Alkylbekova, E.L., Marcus, G.M., Sinner, M.F., Magnani, J.W., Wolf, P.A., Deo, R., Lloyd-Jones, D.M. et al. (2011) Large-scale candidate gene analysis in whites and African Americans identifies IL6R polymorphism in relation to atrial fibrillation: the National Heart, Lung, and Blood Institute's candidate Gene Association resource (CARe) project. Circ. Cardiovasc. Genet., 4, 557–564. 21846873 10.1161/CIRCGENETICS.110.959197 PMC3224824

[55] Hawkins, G.A., Robinson, M.B., Hastie, A.T., Li, X., Li, H., Moore, W.C., Howard, T.D., Busse, W.W., Erzurum, S.C., Wenzel, S.E. et al. (2012) The IL6R variation asp (358) ala is a potential modifier of lung function in subjects with asthma. J. Allergy Clin. Immunol., 130, 510, e511–515. 22554704 10.1016/j.jaci.2012.03.018 PMC3409329

[56] Harrison, S.C., Smith, A.J., Jones, G.T., Swerdlow, D.I., Rampuri, R., Bown, M.J., Aneurysm, C., Folkersen, L., Baas, A.F., de Borst, G.J. et al. (2013) Interleukin-6 receptor pathways in abdominal aortic aneurysm. Eur. Heart J., 34, 3707–3716. 23111417 10.1093/eurheartj/ehs354 PMC3869968

[57] Eyre, S., Bowes, J., Diogo, D., Lee, A., Barton, A., Martin, P., Zhernakova, A., Stahl, E., Viatte, S., McAllister, K. et al. (2012) High-density genetic mapping identifies new susceptibility loci for rheumatoid arthritis. Nat. Genet., 44, 1336–1340. 23143596 10.1038/ng.2462 PMC3605761

[58] Scheller, J., Ohnesorge, N. and Rose-John, S. (2006) Interleukin-6 trans-signalling in chronic inflammation and cancer. Scand. J. Immunol., 63, 321–329. 16640655 10.1111/j.1365-3083.2006.01750.x

[59] Chalaris, A., Schmidt-Arras, D., Yamamoto, K. and Rose-John, S. (2012) Interleukin-6 trans-signaling and colonic cancer associated with inflammatory bowel disease. Dig. Dis., 30, 492–499. 23108305 10.1159/000341698

[60] Enevold, C., Baslund, B., Linde, L., Josephsen, N.L., Tarp, U., Lindegaard, H., Jacobsen, S. and Nielsen, C.H. (2014) Interleukin-6-receptor polymorphisms rs12083537, rs2228145, and rs4329505 as predictors of response to tocilizumab in rheumatoid arthritis. Pharmacogenet. Genomics, 24, 401–405. 24978393 10.1097/FPC.0000000000000071

[61] Ligthart, S., Vaez, A., Vosa, U., Stathopoulou, M.G., de Vries, P.S., Prins, B.P., Van der Most, P.J., Tanaka, T., Naderi, E., Rose, L.M. et al. (2018) Genome analyses of >200,000 individuals identify 58 loci for chronic inflammation and highlight pathways that link inflammation and complex disorders. Am. J. Hum. Genet., 103, 691–706. 30388399 10.1016/j.ajhg.2018.09.009 PMC6218410

[62] Reiner, A.P., Beleza, S., Franceschini, N., Auer, P.L., Robinson, J.G., Kooperberg, C., Peters, U. and Tang, H. (2012) Genome-wide association and population genetic analysis of C-reactive protein in African American and Hispanic American women. Am. J. Hum. Genet., 91, 502–512. 22939635 10.1016/j.ajhg.2012.07.023 PMC3511984

[63] Herder, C., Nuotio, M.L., Shah, S., Blankenberg, S., Brunner, E.J., Carstensen, M., Gieger, C., Grallert, H., Jula, A., Kahonen, M. et al. (2014) Genetic determinants of circulating interleukin-1 receptor antagonist levels and their association with glycemic traits. Diabetes, 63, 4343–4359. 24969107 10.2337/db14-0731 PMC4237993

[64] Ellingsgaard, H., Hauselmann, I., Schuler, B., Habib, A.M., Baggio, L.L., Meier, D.T., Eppler, E., Bouzakri, K., Wueest, S., Muller, Y.D. et al. (2011) Interleukin-6 enhances insulin secretion by increasing glucagon-like peptide-1 secretion from L cells and alpha cells. Nat. Med., 17, 1481–1489. 22037645 10.1038/nm.2513 PMC4286294

[65] van de Veerdonk, F.L., Stoeckman, A.K., Wu, G., Boeckermann, A.N., Azam, T., Netea, M.G., Joosten, L.A., van der Meer, J.W., Hao, R., Kalabokis, V. et al. (2012) IL-38 binds to the IL-36 receptor and has biological effects on immune cells similar to IL-36 receptor antagonist. Proc. Natl. Acad. Sci. USA., 109, 3001–3005. 22315422 10.1073/pnas.1121534109 PMC3286950

[66] Reiner, A.P., Wurfel, M.M., Lange, L.A., Carlson, C.S., Nord, A.S., Carty, C.L., Rieder, M.J., Desmarais, C., Jenny, N.S., Iribarren, C. et al. (2008) Polymorphisms of the IL1-receptor antagonist gene (IL1RN) are associated with multiple markers of systemic inflammation. *Arterioscler*. Thromb. Vasc. Biol., 28, 1407–1412. 10.1161/ATVBAHA.108.167437 PMC2748384 18451331

[67] Hom, G., Graham, R.R., Modrek, B., Taylor, K.E., Ortmann, W., Garnier, S., Lee, A.T., Chung, S.A., Ferreira, R.C., Pant, P.V. et al. (2008) Association of systemic lupus erythematosus with C8orf13-BLK and ITGAM-ITGAX. N. Engl. J. Med., 358, 900–909. 18204098 10.1056/NEJMoa0707865

[68] Jostins, L., Ripke, S., Weersma, R.K., Duerr, R.H., McGovern, D.P., Hui, K.Y., Lee, J.C., Schumm, L.P., Sharma, Y., Anderson, C.A. et al. (2012) Host-microbe interactions have shaped the genetic architecture of inflammatory bowel disease. Nature, 491, 119–124. 23128233 10.1038/nature11582 PMC3491803

[69] Ahluwalia, T.S., Schulz, C.A., Waage, J., Skaaby, T., Sandholm, N., van Zuydam, N., Charmet, R., Bork-Jensen, J., Almgren, P., Thuesen, B.H. et al. (2019) A novel rare CUBN variant and three additional genes identified in Europeans with and without diabetes: results from an exome-wide association study of albuminuria. Diabetologia, 62, 292–305. 30547231 10.1007/s00125-018-4783-z PMC6323095

[70] Apelqvist, J. (2012) Diagnostics and treatment of the diabetic foot. Endocrine, 41, 384–397. 22367583 10.1007/s12020-012-9619-x

[71] Pickrell, J.K., Berisa, T., Liu, J.Z., Segurel, L., Tung, J.Y. and Hinds, D.A. (2016) Detection and interpretation of shared genetic influences on 42 human traits. Nat. Genet., 48, 709–717. 27182965 10.1038/ng.3570 PMC5207801

[72] Mac Rae, C.A. and Vasan, R.S. (2011) Next-generation genome-wide association studies: time to focus on phenotype? Circ. Cardiovasc. Genet., 4, 334–336. 21846867 10.1161/CIRCGENETICS.111.960765 PMC3187849

[73] Dumitrescu, L., Carty, C.L., Franceschini, N., Hindorff, L.A., Cole, S.A., Buzkova, P., Schumacher, F.R., Eaton, C.B., Goodloe, R.J., Duggan, D.J. et al. (2013) Post-genome-wide association study challenges for lipid traits: describing age as a modifier of gene-lipid associations in the population architecture using genomics and epidemiology (PAGE) study. Ann. Hum. Genet., 77, 416–425. 23808484 10.1111/ahg.12027 PMC3796061

[74] Kulminski, A.M., Culminskaya, I., Arbeev, K.G., Arbeeva, L., Ukraintseva, S.V., Stallard, E., Wu, D. and Yashin, A.I. (2015) Birth cohort, age, and sex strongly modulate effects of lipid risk alleles identified in genome-wide association studies. PLoS One, 10, e0136319. 26295473 10.1371/journal.pone.0136319 PMC4546650

[75] Marchini, J., Howie, B., Myers, S., McVean, G. and Donnelly, P. (2007) A new multipoint method for genome-wide association studies by imputation of genotypes. Nat. Genet., 39, 906–913. 17572673 10.1038/ng2088

[76] Howie, B., Fuchsberger, C., Stephens, M., Marchini, J. and Abecasis, G.R. (2012) Fast and accurate genotype imputation in genome-wide association studies through pre-phasing. Nat. Genet., 44, 955–959. 22820512 10.1038/ng.2354 PMC3696580

[77] Servin, B. and Stephens, M. (2007) Imputation-based analysis of association studies: candidate regions and quantitative traits. PLoS Genet., 3, e114. 17676998 10.1371/journal.pgen.0030114 PMC1934390

[78] van der Most, P.J., Vaez, A., Prins, B.P., Munoz, M.L., Snieder, H., Alizadeh, B.Z. and Nolte, I.M. (2014) QCGWAS: a flexible R package for automated quality control of genome-wide association results. Bioinformatics, 30, 1185–1186. 24395754 10.1093/bioinformatics/btt745

[79] Magi, R. and Morris, A.P. (2010) GWAMA: software for genome-wide association meta-analysis. BMC Bioinformatics, 11, 288. 20509871 10.1186/1471-2105-11-288 PMC2893603

[80] Pei, Y.F., Zhang, L., Li, J. and Deng, H.W. (2010) Analyses and comparison of imputation-based association methods. PLoS One, 5, e10827. 20520814 10.1371/journal.pone.0010827 PMC2877082

[81] Willer, C.J., Li, Y. and Abecasis, G.R. (2010) METAL: fast and efficient meta-analysis of genomewide association scans. Bioinformatics, 26, 2190–2191. 20616382 10.1093/bioinformatics/btq340 PMC2922887

[82] Yang, J., Benyamin, B., McEvoy, B.P., Gordon, S., Henders, A.K., Nyholt, D.R., Madden, P.A., Heath, A.C., Martin, N.G., Montgomery, G.W. et al. (2010) Common SNPs explain a large proportion of the heritability for human height. Nat. Genet., 42, 565–569. 20562875 10.1038/ng.608 PMC3232052

[83] Penninx, B.W., Beekman, A.T., Smit, J.H., Zitman, F.G., Nolen, W.A., Spinhoven, P., Cuijpers, P., De Jong, P.J., Van Marwijk, H.W., Assendelft, W.J. et al. (2008) The Netherlands study of depression and anxiety (NESDA): rationale, objectives and methods. Int. J. Methods Psychiatr. Res., 17, 121–140. 18763692 10.1002/mpr.256 PMC6878352

[84] Purcell, S., Neale, B., Todd-Brown, K., Thomas, L., Ferreira, M.A., Bender, D., Maller, J., Sklar, P., de Bakker, P.I., Daly, M.J. et al. (2007) PLINK: a tool set for whole-genome association and population-based linkage analyses. Am. J. Hum. Genet., 81, 559–575. 17701901 10.1086/519795 PMC1950838

[85] Hinrichs, A.S., Karolchik, D., Baertsch, R., Barber, G.P., Bejerano, G., Clawson, H., Diekhans, M., Furey, T.S., Harte, R.A., Hsu, F. et al. (2006) The UCSC genome browser database: update 2006. Nucleic Acids Res., 34, D590–D598. 16381938 10.1093/nar/gkj144 PMC1347506

[86] Danecek, P., Auton, A., Abecasis, G., Albers, C.A., Banks, E., DePristo, M.A., Handsaker, R.E., Lunter, G., Marth, G.T., Sherry, S.T. et al. (2011) The variant call format and VCFtools. Bioinformatics, 27, 2156–2158. 21653522 10.1093/bioinformatics/btr330 PMC3137218

[87] Wang, K., Li, M. and Hakonarson, H. (2010) ANNOVAR: functional annotation of genetic variants from high-throughput sequencing data. Nucleic Acids Res., 38, e164. 20601685 10.1093/nar/gkq603 PMC2938201

[88] Pruitt, K.D., Tatusova, T. and Maglott, D.R. (2005) NCBI reference sequence (RefSeq): a curated non-redundant sequence database of genomes, transcripts and proteins. Nucleic Acids Res., 33, D501–D504. 15608248 10.1093/nar/gki025 PMC539979

[89] Staley, J.R., Blackshaw, J., Kamat, M.A., Ellis, S., Surendran, P., Sun, B.B., Paul, D.S., Freitag, D., Burgess, S., Danesh, J. et al. (2016) Pheno scanner: a database of human genotype-phenotype associations. Bioinformatics, 32, 3207–3209. 27318201 10.1093/bioinformatics/btw373 PMC5048068

