# Supplementary material for: Genome-wide association study of circulating interleukin 6 levels identifies novel loci
Source: Hum Mol Genet. 2021 Jan 30;30(5):393–409. doi: 10.1093/hmg/ddab023 (PMC8098112; doi:10.1093/hmg/ddab023)
Supplement: Supplementary_Material_IL6_GWAS_11012021_ddab023 [file supplementary_material_il6_gwas_11012021_ddab023.docx]

**Supplemental Material**

Genome Wide Association Study of Circulating Interleukin 6 Levels Identifies Novel Loci.

Tarunveer S. Ahluwalia, *et al.*

**CONTENTS**

| **Topic** | **page** |
| --- | --- |
| **Participating Cohorts: Study Description** |  |
| **S1. Discovery cohorts (n=26)**  S1.1 Alspac  S1.2 Amish  S1.3 ARIC  S1.4 BLSA  S1.5 CHS  S1.6 CoLaus  S1.7 FamHS  S1.8 FHS  S1.9 GOLDN  S1.10 HBCS  S1.11 HealthABC  S1.12 InCHIANTI  S1.13 LBC1921  S1.14 LBC1936  S1.15 LLS  S1.16 LURIC  S1.17 MONICA/KORA  S1.18 MrOS Sweden  S1.19 NESDA  S1.20 NTR  S1.21 PROSPER/PHASE  S1.22 RS1  S1.23 Sardinia  S1.24 SHIP  S1.25 TwinsUK  S1.26 YFS | 4 |
| **S2. Replication cohorts (n=12)**  S2.1 BRHS  S2.2 BWHHS  S2.3 CAPS  S2.4 EAS  S2.5 GOYA Male  S2.6 HIFMECH  S2.7 MAS/SMAS  S2.8 NTR2  S2.9 OATS  S2.10 QIMR  S2.11 STANISLAS  S2.12 WHII | 13 |
| **S3. Statistical modeling & Quality Control**  S3.1 Phenotype trait transformation  S3.2 Phenotype data QC method  S3.3 Genotype data QC method  S3.4 Meta-analyses | 17 |
| **S4. CHARGE IL-6 GWAS Analysis Plan** | 18 |
| **Supplementary Figures**  Figure 1 Study design (and GWAS quality control) overview  Figure 2.1 Forrest Plot rs6734238  Figure 2.2 Forrest Plot rs660895  Figure 2.3 Forrest Plot rs4537545 | 20 |
| **References** | |
| **Supplementary Tables: Accompanied Microsoft Excel document** | |
| Table 1. Participating cohorts  Table 2. Participating study characteristics  Table 3. IL-6 Measurement methods (All Cohorts)  Table 4. Genotyping, quality control, and imputation. (Discovery and Replication)  Table 5. IL6 associated genome-wide significant SNPs and summary statistics after discovery meta-analysis Table 6. IL6 associated (p_discovery_<1×10^-5^) SNP results, selected for replication  Table 7. Discovery, replication, and combined meta-analysis summary results for 12 SNPs.  Table 8. Conditional analysis of *IL1R* locus.  Table 9. Imputation Quality (r^2^) for Index SNPs (Discovery and Replication)  Table 10. Known GWAS associations (p < 5 × 10^-8^) of index IL6 SNPs with other traits  Table 11. Known gene expression associations (p < 5 × 10^-8^) of index IL6 SNPs with other traits  Table 12. Acknowledgments and funding statement | |

#

# **Study Description**

**1.0 Discovery Cohorts**

## **1.1 Avon Longitudinal Study of Parents and their Children (ALSPAC) (1)**

ALSPAC is a longitudinal population-based birth cohort that recruited 14,541 pregnant women residents in Avon, the UK with expected dates of delivery 1st April 1991 to 31st December 1992. 14,541 is the initial number of pregnancies for which the mother enrolled in the ALSPAC study and had either returned at least one questionnaire or attended a “Children in Focus” clinic by 19/07/99. Of these initial pregnancies, there was a total of 14,676 fetuses, resulting in 14,062 live births and 13,988 children who were alive at 1 year of age. When the oldest children were approximately 7 years of age, an attempt was made to bolster the initial sample with eligible cases who had failed to join the study originally. As a result, when considering variables collected from the age of seven onwards (and potentially abstracted from obstetric notes) there are data available for more than the 14,541 pregnancies mentioned above. The number of new pregnancies not in the initial sample (known as Phase I enrolment) that are currently represented on the built files and reflecting enrolment status at the age of 18 is 706 (452 and 254 recruited during Phases II and III respectively), resulting in an additional 713 children being enrolled. The phases of enrolment are described in more detail in the cohort profile paper (see footnote 4 below). The total sample size for analyses using any data collected after the age of seven is therefore 15,247 pregnancies, resulting in 15,458 foetuses. Of this total sample of 15,458 foetuses, 14,775 were live births and 14,701 were alive at 1 year of age. A 10% sample of the ALSPAC cohort, known as the Children in Focus (CiF) group, attended clinics at the University of Bristol at various time intervals between 4 to 61 months of age. The CiF group were chosen at random from the last 6 months of ALSPAC births (1432 families attended at least one clinic). Excluded were those mothers who had moved out of the area or were lost to follow-up, and those partaking in another study of infant development in Avon. This cohort is described in detail on the website ([http://www.alspac.bris.ac.uk](http://www.alspac.bris.ac.uk/)) and elsewhere ^(9,10)^. The ALSPAC website contains details of all the data that are available through a fully searchable data dictionary (<http://www.bris.ac.uk/alspac/researchers/data-access/data-dictionary/>). Parental consent and child’s consent was obtained for all measurements made. Ethical approval for the study was obtained from the ALSPAC Ethics and Law Committee and from the UK National Health Service Local Research Ethics Committees. This cohort is described in detail on the website (http://www.alspac.bris.ac.uk) and elsewhere (1).

Blood samples were collected from participants who gave consent for venipuncture during clinical assessment at the age 9 follow-up visit. Venous blood was centrifuged and plasma was frozen at -20C and later transferred to -80C, and were analysed within 3–9 months of blood sampling with no freeze-thaw cycles in between. A valid measure of IL6 levels was obtained from 4129 participants.

## **1.2 Amish Heredity and Phenotype Intervention (HAPI) Heart Study (2)**

The Heredity and Phenotype Intervention (HAPI) Heart Study was initiated in 2002 to measure the cardiovascular response to 4 short-term interventions affecting cardiovascular risk factors and to identify the genetic and environmental determinants of these responses. The interventions were carried out in 868 relatively healthy Amish adults aged 20 years and older who were recruited between 2003 and 2006. Fasting blood samples were collected for measurement of blood chemistries and isolation of DNA for genetic analysis. Serum IL6 was measured in 489 of these subjects.

## **1.3 The Atherosclerosis Risk in Communities (ARIC) study (3)**

The Atherosclerosis Risk in Communities (ARIC) study is a prospective epidemiological study designed to investigate the etiology and predictors of cardiovascular disease. It enrolled 15,792 individuals aged 45–64 years from four US communities (Forsyth County, NC; Jackson, MS; suburbs of Minneapolis, MN; and Washington County, MD) in 1987–89 and followed them for four completed visits in 1990–92, 1993–95, 1996–98, and 2011–13. A detailed description of the ARIC study design and methods is published elsewhere (4). For this study, the analysis was restricted to subjects of European descent. Affymetrix 6.0 array genotypes were obtained in 8,861 self-identified whites: 734 individuals were excluded for the following reasons: 1) discordant with previous genotype data, 2) genotypic sex did not match phenotypic sex, 3) suspected first-degree relative of an included individual based on genome-wide genotype data, 4) genetic outlier (as assessed by average Identity by State (IBS) using PLINK and >8 standard deviations along any of first 10 principal components in EIGENSTRAT after 5 iterations. SNPs without chromosomal location, monomorphic SNPs, SNPs whose genotype frequencies between two freezes differed by p<10^-6^, SNPs with HWE p<10^-6^ or call rate <90% were excluded from analysis. Imputation of ~2.5 million autosomal SNPs in HapMap, with reference to release 22 of the CEU ancestry, was performed using the algorithm implemented in MACH. IL6 levels were measured in 511 participants.

## **1.4 Baltimore Longitudinal Study of Aging (BLSA) (5)**

The Baltimore longitudinal study on Aging (BLSA) study is a population-based study aimed to evaluate contributors of healthy aging in the older population residing predominantly in the Baltimore-Washington DC area 1. Starting in 1958, participants were examined every one to four years depending on their age. Currently there are approximately 1100 active participants enrolled in the study. Blood samples were collected for DNA extraction, and genome-wide genotyping was completed for 1231 subjects using Illumina 550K. The BLSA has continuing approval from the Institutional Review Board (IRB) of Medstar Research Institute.

The analysis was restricted to subjects with European ancestry and each analysis was further adjusted for the top two principal components derived from an EIGENSTRAT analysis utilizing ~10,000 randomly selected SNPs from the 550K SNP panel 2 (6). Genotyping was completed for 477 participants of European ancestry using a call rate of >98.5% without sex discrepancy based on homozygosity rates and with CRP data. 501,704 autosomal SNPs passed quality control (completeness>99%, MAF >1%, HWE >10-4) were used to for imputation. Imputation of ~39M SNPs was conducted using the 1000G Phase I Integrated Release Version 3 Haplotypes as reference with minimac3 (7).

## **1.5 Cardiovascular Health Study (CHS) (8)**

The CHS is a population-based cohort study of risk factors for CHD and stroke in adults ≥65 years conducted across four field centers. The original predominantly Caucasian cohort of 5,201 persons was recruited in 1989-1990 from random samples of the Medicare eligibility lists; subsequently, an additional predominantly African-American cohort of 687 persons were enrolled for a total sample of 5,888.

Blood samples were drawn from all participants at their baseline examination and DNA was subsequently extracted from available samples. Genotyping was performed at the General Clinical Research Center’s Phenotyping/Genotyping Laboratory at Cedars-Sinai among CHS participants who consented to genetic testing and had DNA available using the Illumina 370CNV BeadChip system (for European ancestry participants, in 2007) or the Illumina HumanOmni1-Quad_v1 BeadChip system (for African-American participants, in 2010).

European ancestry participants were excluded from the GWAS study sample due to the presence at study baseline of coronary heart disease, congestive heart failure, peripheral vascular disease, valvular heart disease, stroke or transient ischemic attack or lack of available DNA. Beyond laboratory genotyping failures, participants were excluded if they had a call rate<=95% or if their genotype was discordant with known sex or prior genotyping (to identify possible sample swaps). Blood was drawn in the morning after an overnight fast. Samples were promptly centrifuged at 3000g for 10 minutes at 4°C. Aliquots of plasma were stored in a central laboratory at –70°C. IL6 was measured in 3028 participants.

**1.6 Cohorte Lausannoise (CoLaus) (9)** This prospective population cohort includes 6,733 Caucasians aged 35-75 years and randomly selected from the general population in Lausanne, Switzerland. These individuals underwent a detailed phenotypic assessment, and were genotyped using the Affymetrix Mapping 500K array. 5’612 samples passed genotyping quality control. Recruitment took place between June 2003 and May 2006. The institutional review boards of the University of Lausanne approved this study, and written consent was obtained from all participants. Il-6 levels were measured using a multiplexed particle-based flow cytometric cytokine assay (Milliplex kits, Millipore, Zug, Switzerland).

## **1.7 Family Heart Study (FamHS) (10)** The Family Heart Study (FamHS;<https://dsgweb.wustl.edu/PROJECTS/MP1.html> ) is a longitudinal and population-based family study designed to investigate the determinants of cardiovascular disease and coronary heart disease (CHD). Approximately 6,000 European ancestry (EA) subjects were selected at random (588 families) or ascertained for family history of CHD (656 families) using information collected in the parent studies—Framingham Heart Study (Framingham, MA, USA), the Utah Health Family Tree Study (Salt Lake City, UT, USA) or the Atherosclerosis Risk in Communities Study (Minneapolis Suburbs, MN, USA and Forsyth County, NC, USA). In the first clinical visit (in 1992), a broad range of phenotypes were assessed in the general domains of CHD, including atherosclerosis, lipids, glucose metabolism, blood pressure, and adipose and anthropometry, inflammation. Approximately 8 years later, study participants belonging to the largest pedigrees were invited for a second clinical visit (2002-2004). A total of 2,756 subjects in 510 extended families were studied. Measurements of the most important CHD risk factors were assessed, and also CT images were read for coronary and aortic calcification, liver attenuation and abdominal visceral and subcutaneous fat. IL6 measures were available for 1304 individuals. The FamHS genotype data included three genotype platforms, Illumina 550K, Illumina 610k, and Illumina 1M. For imputation process, we used Phase II CEU HapMap population (release 22, build 36)http://hapmap.ncbi.nlm.nih.gov/downloads/phasing/2007-08_rel22/phased/). A total of 1304 EA subjects, from the first clinic visit with IL6 measures available were included in the current study.

## **1.8 Framingham Heart Study (FHS)** (11) The Framingham Offspring Study is a community-based observational study initiated in 1948. Three generations of participants have been enrolled and followed at 2-8 year intervals. The Original Cohort consists of 5209 men and women. The Offspring Cohort was recruited in 1971, and consists of 5124 participants. They are the children of the Original Cohort and their spouses (12). The Third Generation cohort was recruited in 2002, and consists of 4095 participants who are mostly children of the Offspring Cohort (13). All study participants have given informed consent, and the study protocol was approved by the Boston University Medical Center Review Board. IL6 was measured in the seventh examination for the Offspring Cohort and the first examination for the Third Generation Cohort. Fasting serum samples was collected from participants after fasting for more than eight hours. A total of 6858 participants from the Offspring and Third generation cohorts had both IL6 measures and good quality genotypes available and were included in the analyses.

## **1.9 Genetics of Lipid Lowering Drugs and Diet Network (GOLDN) (14)** The National Heart, Lung, and Blood Institute GOLDN study was designed to identify genetic determinants to lipid-raising (ingesting a high-fat milkshake) and lipid-lowering (daily treatment with 160mg of fenofibrate for 3 weeks) interventions. The study included families of European descent with at least 2 siblings, who were recruited from the genetically homogeneous Minneapolis and Salt Lake City centers of the National Heart, Lung, and Blood Institute Family Heart study. Participants discontinued the use of lipid-lowering agents for at least 4 weeks, fasted for at least 8 hours, and abstained from alcohol for at least 24 hours prior to study visits. The study protocol was approved by Institutional Review Boards at the University of Minnesota, University of Utah, and Tufts University/New England Medical Center. Of the original 1327 participants, 821 had both genotype and C-reactive phenotype measurements and were included in the analysis. IL6 was measured in 821 individuals.

## **1.10 Helsinki Birth Cohort Study (HBCS) (15, 16)** The Helsinki Birth Cohort Study (HBCS) includes 8,760 individuals born between the years 1934-44 at the Women’s Hospital of the Helsinki University Central Hospital. Between 2001 and 2004, a randomly selected sample of 928 males and 1,075 females participated in a clinical follow-up study with a focus on cardiovascular, metabolic and reproductive health, cognitive function and depressive symptoms. During this visit blood was drawn for the IL6 measurement. Venous blood was centrifuged after coagulation and serum was frozen immediately at -20C and after about one month at -70C until measurement. There were 1,716 women and men (43% men) with valid genotype and phenotype data. The mean age of the participants was 61.5 years (SD=2.9). Detailed information on the selection of the HBCS participants and on the study design can be found elsewhere (15, 16). Research plan of the HBCS was approved by the Institutional Review Board of the National Public Health Institute and all participants have signed an informed consent.

## **1.11 Health Aging and Body Composition Study (Health ABC)** (17) The Health ABC study is a prospective cohort study investigating the associations between body composition, weight-related health conditions, and incident functional limitation in older adults. Health ABC enrolled well-functioning, community-dwelling white (n=1794) men and women aged 70-79 years between April 1997 and June 1998. Participants were recruited from a random sample of all white and black Medicare eligible residents in the Pittsburgh, PA, and Memphis, TN, metropolitan areas. Of 3,075 participants at baseline, 1597 Caucasians had both genotype and phenotype data available for the current study. Genomic DNA was extracted from buffy coat collected using PUREGENE® DNA Purification Kit during the baseline exam. Genotyping was performed by the Center for Inherited Disease Research (CIDR) using the Illumina Human1M-Duo BeadChip system. Samples were excluded from the dataset for the reasons of sample failure, genotypic sex mismatch, and first-degree relative of an included individual based on genotype data. Genotyping was successful for 1,151,215 SNPs in 2,802 unrelated individuals (1663 Caucasians and 1139 African Americans). Imputation was done for the autosomes using the MACH software version 1.0.16. SNPs with minor allele frequency ≥ 1%, call rate ≥97% and HWE p≥10-6 were used for imputation. HapMap II phased haplotypes were used as reference panels. For EAs, genotypes were available on 914,263 high quality SNPs for imputation based on the HapMap CEPH reference panel (release 22, build 36). For AAs, genotypes were available on 1,007,948 high quality SNPS for imputation based on a 1:1 mixture of the CEPH:Yoruban (YRI) reference panel (release 21, build 36). A total of 2,543,887 in EAs and 1,958,375 SNPs in AAs are available for analysis.

**1.12 InCHIANTI study (18)**The InCHIANTI study is a population-based epidemiological study aimed at evaluating the factors that influence mobility in the older population living in the Chianti region in Tuscany, Italy. Briefly, 1616 residents were selected from the population registry of Greve in Chianti (a rural area: 11,709 residents with 19.3% of the population greater than 65 years of age), and Bagno a Ripoli (Antella village near Florence; 4,704 inhabitants, with 20.3% greater than 65 years of age). The participation rate was 90% (n=1453), and the subjects ranged between 21-102 years of age. Overnight fasted blood samples were for genomic DNA extraction, and measurement of IL6. Illumina Infinium HumanHap 550K SNP arrays were used for genotyping(20). The study protocol was approved by the Italian National Institute of Research and Care of Aging Institutional Review and Medstar Research Institute (Baltimore, MD). 1208 individuals who passed quality control with a sample call rate >97%, heterozygosity rates > 0.3 and correct sex specification and with CRP data. 495,343 autosomal SNPs that passed quality control (MAF>1%, completeness >99%, HWE > 10-4) were used for imputation. Imputation of ~39M SNPs was conducted using the 1000G Phase I Integrated Release Version 3 Haplotypes as reference with minimac3.

## **1.13 Lothian Birth Cohorts (LBC) 1921 (19-21)** The Lothian Birth Cohorts of 1921 and 1936 are two longitudinal studies of ageing. They derive from the Scottish Mental Surveys of 1932 and 1947, respectively, when nearly all 11 year old children in Scotland completed a test of general cognitive ability(23). Survivors living in the Lothian area of Scotland were recruited in late-life at mean age 79 for LBC1921 (n=550) and mean age 70 for LBC1936 (n=1,091). Follow-up has taken place at ages 79, 83, 87, and 90 in LBC1921.

Collected data include genetic information, longitudinal epigenetic information, longitudinal brain imaging (LBC1936), and numerous blood biomarkers, anthropomorphic and lifestyle measures.

Ethics permission for the study was obtained from the Multi-Centre Research Ethics Committee for Scotland (MREC/01/0/56) and from Lothian Research Ethics Committee (LBC1936: LREC/2003/2/29 and LBC1921: LREC/1998/4/183). The research was carried out in compliance with the Helsinki Declaration. All subjects gave written, informed consent. At the first LBC1936 visit and the third LBC1921 visit, non-fasting blood samples were collected. The samples were genotyped at the Wellcome Trust Clinical Research Facility Genetics Core, Western General Hospital, Edinburgh. The Illumina Human 610-Quad v1.0 array was used and genotype calling algorithm used was Illumina GenomeStudio. Genotype and phenotype data (IL6) was available in n=166 (LBC1921) participants.

**1.14 Lothian Birth Cohorts (LBC) 1936 (19-21)**The Lothian Birth Cohorts of 1921 and 1936 are two longitudinal studies of ageing. They derive from the Scottish Mental Surveys of 1932 and 1947, respectively, when nearly all 11 year old children in Scotland completed a test of general cognitive ability(23). Survivors living in the Lothian area of Scotland were recruited in late-life at mean age 79 for LBC1921 (n=550) and mean age 70 for LBC1936 (n=1,091). Follow-up has taken place at ages 70, 73, and 76 in LBC1936. Collected data include genetic information, longitudinal epigenetic information, longitudinal brain imaging (LBC1936), and numerous blood biomarkers, anthropomorphic and lifestyle measures. Ethics permission for the study was obtained from the Multi-Centre Research Ethics Committee for Scotland (MREC/01/0/56) and from Lothian Research Ethics Committee (LBC1936: LREC/2003/2/29 and LBC1921: LREC/1998/4/183). The research was carried out in compliance with the Helsinki Declaration. All subjects gave written, informed consent. At the first LBC1936 visit and the third LBC1921 visit, non-fasting blood samples were collected. The samples were genotyped at the Wellcome Trust Clinical Research Facility Genetics Core, Western General Hospital, Edinburgh. The Illumina Human 610-Quad v1.0 array was used and genotype calling algorithm used was Illumina GenomeStudio. Genotype and phenotype data (IL6) was available in n=759 (LBC1936) participants.

## **1.15 Leiden Longevity Study (LLS)** (22) For the Leiden Longevity Study, long-lived siblings of European descent were recruited together with their offspring (N=1671) and the partners of the offspring (N=744) (24, 25). For this IL6 GWAS the joint sample of offspring and controls (N_total_ = 1798) having available both genetic and Il6 plasma levels, were analysed. We analyze the two groups as 1 cohort of middle aged people in which we have to account for sibling relatedness. Mean age is 59.19 years (SD=6.8), mean BMI =25.4 (SD=3.6). We have GWAS data available on 1585 offspring (Illumina 660 Quad) and 735 controls (265 Illumina 660 Quad and 470 Illumina 770 OmniExpress.

## **1.16 Ludwigshafen Risk and Cardiovascular Health (LURIC) Study** (23) The LURIC study included 3,316 Caucasians hospitalized for coronary angiography between 1997 and 2000 at a tertiary care centre in south-western Germany. Clinical indications for angiography were chest pain or a positive non-invasive stress test suggestive of myocardial ischemia. To limit clinical heterogeneity, individuals suffering from acute illnesses other than acute coronary syndrome, chronic non-cardiac diseases and a history of malignancy within the five past years were excluded. The study was approved by the ethics committee at the "Landesärztekammer Rheinland-Pfalz" and was conducted in accordance with the "Declaration of Helsinki”. Informed written consent was obtained from all participants. Information on vital status was obtained from local registries. Death certificates and medical records of local hospitals and autopsy data were reviewed independently by two experienced clinicians who were blinded to patient characteristics and who classified the causes of death. In cases of disagreement or uncertainty concerning the coding of a specific cause of death the decision was made by a principal investigator (W.M.). Fasting blood samples were obtained by venipuncture in the early morning. A total of 604 individuals had genotype and phenotype data for the current study.

## **1.17 Monitoring of Trends and Determinants in Cardiovascular Disease (MONICA)/ Cooperative Health Research in the Region of Augsburg (KORA) F3/F4 study (24)** The MONICA/KORA Augsburg Study consisted of a series of independent population-based epidemiological surveys of participants living in the region of Augsburg, Southern Germany. All survey participants are residents of German nationality identified through the registration office. The study was approved by the ethics committee of the Bavarian Medical Association, and informed written consent was obtained from all participants.

## The presented data with HapMap data were derived from the third population-based Monitoring of Trends and Determinants in Cardiovascular Disease (MONICA)/ Cooperative Health Research in the Region of Augsburg (KORA) surveys S3. This cross-sectional survey covering the city of Augsburg (Germany) and two adjacent counties was conducted in 1994/95 with 4,856 individuals aged 25 to 74 years as part of the WHO MONICA study. In a follow-up examination of S3 conducted in 2004/05 (MONICA/KORA F3), a number of 3,006 subjects participated. All participants underwent standardized examinations including blood withdrawals for plasma and DNA. For the MONICA/KORA genome-wide association study, a number of 1,644 was selected from F3 samples. After excluding subjects with no IL6 measurements available or IL6 extreme values, the final populations for the MONICA/KORA data sets comprised 1,625 (S3/F3) subjects. Genotyping for F3 was performed using Affymetrix 500K Array Set consisting of two chips (Sty I and Nsp I). Genotypes were determined using BRLMM clustering algorithm (Affymetrix 500K Array Set). For quality control purposes, we applied a positive control and a negative control DNA every 48 samples F3. On chip level only subjects with overall genotyping efficiencies of at least 93% were included. In addition, the called gender had to agree with the gender in the MONICA/KORA study database. SNPs were excluded from analysis when monomorphic (MAF < 0.01). Imputation of genotypes was performed using maximum likelihood method with the software MACH v1.0.9. Blood was collected in fasting subjects without stasis and kept at 4°C until centrifugation.

## **1.18 The Osteoporotic Fractures in Men (MrOS) Sweden (MrOS Sweden) study (25)** The Osteoporotic Fractures in Men (MrOS) study is a multicenter, prospective study including older men in Sweden, Hong Kong, and the United States. The Swedish MrOS cohort (n= 3014) consists of three sub-cohorts from three different Swedish cities (n=1005 in Malmö, n=1010 in Gothenburg, and n=999 in Uppsala). In the present study, only subjects from Gothenburg were included. The study subjects (men 69–80 yr of age) were randomly identified using national population registers. A total of 45% of the subjects who were contacted participated in the study. All participants are of European ancestry. To be eligible for the study, the subjects had to be able to walk without aids. There were no other exclusion criteria. The study was approved by the ethics committee at the University of Gothenburg. Informed consent was obtained from all study participants. 938 individuals participated in the current study.

## **1.19 Netherlands Study of Depression and Anxiety (NESDA) Study (26)** The Netherlands Study of Depression and Anxiety (NESDA) is an ongoing cohort study into the long-term course and consequences of depressive and anxiety disorders. In 2004-2007 participants aged 18 to 65 years were recruited from the community (19%), general practice (54%) and secondary mental health care (27%), reflecting therefore various settings and developmental stages of psychopathology in order to obtain a full and generalizable picture of the course of psychiatric disorders. A total of 2,981 participants were included, consisting of persons with a current or past depressive and/or anxiety disorder and healthy controls. The research protocol was approved by the ethical committee of participating universities, and all respondents provided written informed consent. Markers of inflammation were assessed at the baseline NESDA measurement. Fasting blood samples of NESDA participants were obtained in the morning around 0800 hours and kept frozen at -70 ^○^C. A total of 1746 participants had genotype and phenotype data for the current analyses.

## **1.20 Netherlands Twin Register (NTR) (27)** As part of a Netherlands Twin Register (NTR) biobank project, 9,530 participants from 3,477 families were visited at home between January 2004 and July 2008 for collection of blood samples. Visits were scheduled between 7:00 and 10:00 am and fertile women were bled on day 2–4 of the menstrual cycle, or in their pill-free week. Fertile women were bled on day 2–4 of the menstrual cycle, or in their pill-free week. Body composition was measured and information about physical health and lifestyle (e.g. smoking and drinking behavior, physical exercise, medication use) was obtained. For more detailed information about the methodology of the NTR Biobank study (27). The NTR studies were approved by the Central Ethics Committee on Research involving human subjects of the VU University Medical Center, Amsterdam, an Institutional Review Board certified by the US Office of Human Research Protections (IRB number IRB-2991 under Federal wide Assurance-3703; IRB/institute codes, NTR 03-180). All subjects provided written informed consent. Valid GWA data were available for 3152 individuals to participate in the discovery IL6 GWAS.

## **1.21 PROspective Study of Pravastatin in the Elderly at Risk (PROSPER/PHASE) (28)** PROSPER was a prospective multicenter randomized placebo-controlled trial to assess whether treatment with pravastatin diminishes the risk of major vascular events in elderly. Between December 1997 and May 1999, we screened and enrolled subjects in Scotland (Glasgow), Ireland (Cork), and the Netherlands (Leiden). Men and women aged 70-82 years were recruited if they had pre-existing vascular disease or increased risk of such disease because of smoking, hypertension, or diabetes. A total number of 5,804 subjects were randomly assigned to pravastatin or placebo. A large number of prospective tests were performed including Biobank tests and cognitive function measurements. All measurements were performed on previously un-thawed baseline samples stored at -80°C. A whole genome wide screening has been performed in the sequential PHASE project with the use of the Illumina 660K BeadChip. Of 5,763 subjects DNA was available for genotyping. Genotyping was performed with the Illumina 660K BeadChip, after QC (call rate <95%) 5,244 subjects and 557,192 SNPs were left for analysis. These SNPs were imputed to 2.5 million SNPs based on the HAPMAP built 36 with MACH imputation software. A total of 5130 individuals participated in the current IL6 GWAS study.

## **1.22 Rotterdam Study (RS1) (29)** The Rotterdam Study is a prospective population-based cohort study started in 1990 to study determinants of common diseases. The first cohort included 7,983 inhabitants aged 55 years and older that were living in the well-defined district Ommoord in Rotterdam, the Netherlands. The baseline examinations took place between 1990 and 1993, after which additional examinations at the research center took place every 3-4 years. In 2000, the Rotterdam study was extended with a second cohort of 3011 individuals who reached the age of 55 and persons 55 years or older that moved into the research area. The baseline examinations of the second cohort took place from begin 2000 until the end of 2001. The third cohort was initiated in 2006 and included 3932 individuals aged 45 years and older. Baseline examinations for the third cohort were finished end 2008. All participants are of European ancestry based on their self-report. The Rotterdam Study has been approved by the medical ethics committee according to the Population Study Act Rotterdam Study, executed by the Ministry of Health, Welfare and Sports of the Netherlands. A written informed consent was obtained from all participants.

For the first cohort, non-fasting serum samples were collected at the baseline center visit. The samples were immediately put on ice and were processed within 30 minutes, after which they were kept frozen at -20 °C until the measurement of IL6 in 2003-2004. A total of 599 individuals with genotype and phenotype data participated.

**1.23 SardiNIA study** The SardiNIA (30) Study is a longitudinal population-based cohort study started in 2001 to study quantitative traits of biomedical relevance with a special emphasis on those influencing aging. The study, now in its 14th year and in its fourth phase, collected the longitudinal information on more than 1000 quantitative traits, including inflammatory markers and immuno-related traits. In a first survey, the project recruited individuals from four towns in the Lanusei Valley (east-central Sardinia) and assessed 98 quantitative traits including over 62% of the eligible population living in the region (age 14-102 years), and at least 96% of the initial cohort have all grandparents born in the same province. The initial group of 6,148 individuals included 4,933 phenotyped sib pairs, 4,266 phenotyped parent-child pairs, >4,069 phenotyped cousin pairs, and >6,459 phenotyped avuncular pairs. A total of n= 4621 individuals participated in the IL6 GWAS. A written informed consent was obtained from all participants.

## **1.24 Study of Health in Pomerania (SHIP) (31, 32)** The Study of Health in Pomerania (SHIP) is a population-based project in West Pomerania, the north-east area of Germany. A sample from the population aged 20 to 79 years was drawn from population registries. First, the three cities of the region (with 17,076 to 65,977 inhabitants) and the 12 towns (with 1,516 to 3,044 inhabitants) were selected, and then 17 out of 97 smaller towns (with less than 1,500 inhabitants), were drawn at random. Second, from each of the selected communities, subjects were drawn at random, proportional to the population size of each community and stratified by age and gender. Only individuals with German citizenship and main residency in the study area were included. Finally, 7,008 subjects were sampled, with 292 persons of each gender in each of the twelve five-year age strata. In order to minimize drop-outs by migration or death, subjects were selected in two waves. The net sample (without migrated or deceased persons) comprised 6,267 eligible subjects. Selected persons received a maximum of three written invitations. In case of non-response, letters were followed by a phone call or by home visits if contact by phone was not possible. The SHIP population finally comprised 4,308 participants (corresponding to a final response of 68.8%). Blood samples were taken from the cubital vein of mostly non-fasting participants in the supine position. Serum aliquots were prepared for immediate analysis and for storage at -80 °C in the Integrated Research Biobank (Liconic, Liechtenstein). 1327 individuals with genotype and phenotype data participated.

## **1.25 TwinsUK (33)** Twins UK comprises unselected, mostly female volunteers ascertained from the general population through national media campaigns in the UK. Zygosity was determined by standardized questionnaire and confirmed by DNA fingerprinting. Individuals were genome-wide genotyped using the Infinium assay (Illumina, San Diego, USA) across three fully compatible SNP arrays. The genotyping and quality control of these data have been described elsewhere. The genotypes were next imputed using IMPUTE version 0.3.2 with Phase II CEU HapMap data (release 22, build 36) as the reference panel. Serum IL-6 was measured using the bead-based high sensitivity human cytokine kit (HSCYTO-60SK, Linco-Millipore) according to the manufacturer’s protocol. Each sample was assayed in duplicate and standards were included in each plate. Sensitivity values for the kit was 0.10 pg/ml. A total of n=1103 individuals had data on IL6 and genotypes available for participation. Written informed consent was obtained from all participants before they entered the studies, which were approved by the local research ethics committee.

## **1.26 Young Finns Study (YFS) (34)** The YFS is a population-based follow up-study started in 1980. The main aim of the YFS is to determine the contribution made by childhood lifestyle, biological and psychological measures to the risk of cardiovascular diseases in adulthood. In 1980, over 3,500 children and adolescents all around Finland participated in the baseline study. The follow-up studies have been conducted mainly with 3-year intervals. The latest 27-year follow-up study was conducted in 2007 (ages 30-45 years) with 2,204 participants. The study was approved by the local ethics committees (University Hospitals of Helsinki, Turku, Tampere, Kuopio and Oulu) and was conducted following the guidelines of the Declaration of Helsinki. All participants gave their written informed consent. Genomic DNA was extracted from peripheral blood leukocytes using a commercially available kit and Qiagen BioRobot M48 Workstation according to the manufacturer’s instructions (Qiagen, Hilden, Germany). Genotyping was done for 2,556 samples using custom build Illumina Human 670k BeadChip at Welcome Trust Sanger Institute. Genotypes were called using Illuminus clustering algorithm. Fifty-six samples failed Sanger genotyping pipeline QC criteria (i.e., duplicated samples, heterozygosity, low call rate, or Sequenom fingerprint discrepancy). From the remaining 2,500 samples one sample failed gender check, three was removed due to low genotyping call rate (< 0.95) and 54 samples for possible relatedness (pi-hat > 0.2) . 11,766 SNPs were excluded based on HWE test (p = 1e-06), 7,746 SNPs failed missingness test (call rate < 0.95) and 34,596 SNPs failed frequency test (MAF < 0.01). After quality control there were 2,443 samples and 546,677 genotyped SNPs available for further analysis. Genotype imputation to HapMap II reference was performed using MACH 1.0 and HapMap II CEU (release 22) samples as reference. Imputation to 1000 Genomes reference was performed using SHAPEIT v1 for haplotype phasing and IMPUTE2 and 1000 Genomes March 2012 haplotypes for genotype imputation.

**2. Replication studies**

## **2.1 British Regional Heart Study (BRHS), (35, 36)** The British Regional Heart Study (BRHS) is a prospective cohort study of CVD involving 7735 middle-aged men (40–59 years) selected in 1978–1980 from the age–sex registers of one local primary care centre in 24 British towns. The 24 towns were selected to represent the variation in CVD across the UK. The general practice selected in each town was required to have a social class distribution representative of the men of that town. The men were selected at random from age-sex registers; no attempt was made to exclude subjects with cardiovascular problems and there was a 78% response rate. Research nurses administered a questionnaire and completed an examination of each man, including an electrocardiogram.

In 1998–2000, an average of 20 years after the initial recruitment, 4252 surviving participants (77% response rate) aged 60–79 years who were resident in the UK attended a physical examination during which nurses took a fasting blood sample on one occasion for each participant. The men were asked to fast for a minimum of 6 hours, during which they were instructed to drink only water. The blood samples were collected between 08:00 and 19:00 hours and then assayed for a range of biochemical and haematological markers.

IL-6 was assayed using a high-sensitivity ELISA (R&D Systems, Oxford, UK). Intra and inter assay coefficients of variation was 7.5% and 8.9%, respectively. Of the 4,252 participants, DNA was extracted for N=3,945; the metabochip typing was carried out on a nested case-control subsample of 2,453. The current study included 2,301 men with complete IL6 and genotype measures available.

## **2.2 British Women’s Heart and Health Study (BWHHS) (37, 38)** The British Women's Heart and Health Study (BWHHS) is a prospective cohort study of 4,286 women who were recruited between March 1999 and April 2001 from 23 towns across Britain. Women were 60 to 79 at recruitment. These women have been followed-up over a median of 4.6 years by flagging with the NHS central register for mortality data and two-yearly review of their medical records. Participants gave written informed consent and the study was approved by the national health service multi-centre research ethics committee.Venous blood samples were taken after a minimum 8 h fast. The aliquots were then snap frozen on dry ice as required and placed in the freezer at −20°C. On completion of each two week block of fieldwork these samples were transferred for long term storage at −80°C. IL-6 was assayed using a high sensitivity ELISA assay (R&D systems, Oxford, UK). Inter- and intra-assay coefficients of variation were 7.5% and 8.9%, respectively. The current study included 1,904 women with complete IL6 and genotype measures available.

## **2.3 Caerphilly Prospective Study (UCLEB – CAPS) (39, 40)** CaPS is a cohort of men born between 1920 and 1939 who were recruited between 1979 and 1983 from Caerphilly and adjacent villages to take part in a prospective cohort study with an initial focus on cardiovascular disease, though as the cohort got older a variety of age-related traits were added. At ages 45-59 years, 2512 men (response rate of 89%) were seen and followed up at phase 2 (1984-1988), phase 3 (1989-1993), phase 4 (1993-1996) and phase 5 (2002-2004). An additional 447 men of similar age were recruited at phase 2 who had moved into the defined area. The genotyping for all UCLEB Consortium cohorts has been described previously (40). There were 704 individuals as part of the current study with genotype and phenotype measures.

## **2.4 Edinburgh Artery Study (EAS) (41)**

Edinburgh Artery Study (EAS). At baseline (August 1987-September 1988), an age-stratified random sample of men and women, aged 55–74 years, was selected from the age-sex registers of ten general practices with catchment populations spread geographically and socioeconomically throughout the city of Edinburgh. Subjects were excluded if they were unfit to participate (e.g. due to severe mental illness or terminal disease). These exclusions were replaced by other randomly sampled subjects. The study population is almost exclusively European. DNA was extracted at 5 years follow-up. Physical examinations were performed by specially trained research nurses using standardised operating procedures. The quality of clinical measurements were checked before and during the study by repeat measurements taken intermittently by the study coordinator. Individual observer measurements were assessed for drift. Blood assays were performed in accredited laboratories using international standards. Subjects have been followed up for 20 years for cardiovascular events, using repeat self-reporting questionnaires, record linkage for hospitalisations and deaths, and validation of events against pre-specified criteria through searching of hospital and GP notes. Comprehensive clinical examination was repeated at 5 and 12 years after commencement of the study, resulting in repeat measurements of several key variables.

## **2.5 Genetics of Overweight Young Adults (GOYA) Male (42, 43)** The GOYA (Male) cohort is a longitudinal case-cohort (obese, non-obese) study comprising a randomly (1%) selected control group and all extremely overweight men identified among 362,200 Caucasian men examined at the mean age of 20 years at the draft boards in Copenhagen and its surrounding areas during 1943–1977. Obesity was defined as 35% overweight relative to a local standard in use at the time (mid 1970’s), corresponding to a BMI ≥31.0 kg/m^2^, which proved to be above the 99th percentile. All of the obese and 50% of the random sampled controls, who were still living in the region, were invited to a follow-up survey in 1992–94 at the mean age of 46 years, at which time the blood samples were taken and genotyping were performed for a total of 673 extremely overweight and 792 controls (30) With a sampling fraction of 0.5% (50% of 1%), the controls represent about 158,000 men among whom the case group was the most obese. A subset of the GOYA (males) was followed up, that formed part of the ADIGEN (acronym for ADIposity GENetics) study where they were deeply phenotyped with additional biochemistry measures including IL6. Blood samples were obtained in the morning after an overnight 12-hour fast, IL6 was measured in 318 participants.

**2.6 The Hypercoagulability and Impaired Fibrinolytic function MECHanisms predisposing to myocardial infarction study (HIFMECH) (44)** The HIFMECH cohort comprises male, Caucasian survivors of myocardial infarction (MI) (excluding patients with familial hypercholesterolaemia and type 1 diabetes mellitus) aged under 60 years and healthy age matched controls, ascertained from four regional centres: Stockholm, Sweden and London, UK representing the North, and Marseille, France and San Giovanni Rotondo, Italy representing the South of Europe. Consecutive patients were recruited between 3–6 months following MI and matched for age with population-based healthy subjects in each geographical region. Participants completed a questionnaire detailing past medical history, current medication, smoking status and alcohol consumption. Patients and controls were examined in parallel during the morning following an overnight fast. Weight, height, systolic and diastolic blood pressures were determined. In total, 413 control subjects with complete genotype and phenotype information were included in this study.

## **2.7 Sydney Memory and Ageing Study (SMAS) (45)** This longitudinal community-based study recruited adults aged 70 -90 years from the compulsory electoral rolls of Sydney, Australia. Participants were excluded for any of the following: limited English, a medical/psychological condition that would prevent them from completing their assessments, dementia diagnosis, psychotic symptoms or diagnosis of schizophrenia/bipolar disorders, multiple sclerosis, motor neuron disease, developmental disability, a progressive malignancy. Written informed consent was obtained from all participants and the study was approved by the relevant ethics committees (University of New South Wales, South Eastern Sydney and Illawarra Area Health Service). A comprehensive assessment including collection of demographic, medical history, physical function and cognitive testing was undertaken. Fasting blood samples were also collected for biochemistry and genetic testing. At baseline, there were 1037 participants with a mean age of 78.84 years and 55.2% were women. 847 individuals with complete genotype and phenotype data participated in the current study.

## **2.8 Netherlands Twin Register (NTR2) (27)** As part of a Netherlands Twin Register (NTR) biobank project, 9,530 participants from 3,477 families were visited at home between January 2004 and July 2008 for collection of blood samples. Visits were scheduled between 7:00 and 10:00 am and fertile women were bled on day 2–4 of the menstrual cycle, or in their pill-free week. Fertile women were bled on day 2–4 of the menstrual cycle, or in their pill-free week. Body composition was measured and information about physical health and lifestyle (e.g. smoking and drinking behavior, physical exercise, medication use) was obtained. For more detailed information about the methodology of the NTR Biobank study, see (27). The NTR studies were approved by the Central Ethics Committee on Research involving human subjects of the VU University Medical Center, Amsterdam, an Institutional Review Board certified by the US Office of Human Research Protections (IRB number IRB-2991 under Federal wide Assurance-3703; IRB/institute codes, NTR 03-180). All subjects provided written informed consent. Valid GWA data were available for 6560 individuals at the time of the IL6 GWAS replication analyses. 3152 Individuals had participated in the discovery analyses whereas 3322 additional individuals participated in the replication analyses.

## **2.9 Older Australian Twin Study (OATS) (46)** Twins aged 65 years and over were recruited from Twin Registry Australia and also via a recruitment drive. Criteria for inclusion into OATS included a consenting co-twin, ability to consent and residence in the three eastern Australian states. Exclusion criteria included current diagnosis of malignancy or insufficient English to complete the assessment. Written informed consent was obtained from all participants and the study was approved by the relevant ethics committees (Australian Twin Registry, University of New South Wales, University of Melbourne, Queensland Institute of Medical Research and the South Eastern Sydney and Illawarra Area Health Service). A comprehensive assessment including collection of demographic, medical history, physical function and cognitive testing was undertaken. Fasting blood samples were also collected for biochemistry and genetic testing. At baseline, there were 623 participants with a mean age of 70.77 years and 65.2% of the sample were women. 372 individuals with complete genotype and phenotype data participated in the current study.

## **2.10 QIMR Berghofer Medical Research Institute, Asthma and Allergy Study (QIMR) (47)**

Serum and DNA samples were available for 360 unrelated asthmatics (mean age 27, range 7 to 73; 54% female) who participated in the 1995–1998 Asthma and Allergy study, as described in detail elsewhere (47). IL-6 levels in serum were available for a subset of 325 individuals, obtained using a Quantikine ELISA assay (Sapphire Bioscience). DNA samples were genotyped with the Illumina 610K array, and genomic coverage expanded by imputing 5.8 million variants using the 1000 Genomes Project reference panel and Impute2.

**2.11 Suivi Temporaire Annuel Non Invasif de la Santé des Lorrains Assurés Sociaux study (STANISLAS) (48)**

The STANISLAS Family Study is a 10-year longitudinal study that includes 1006 families residing in Vandoeuvre-lès-Nancy (France), recruited between 1993 and 1995. Two-parent families (n=4455) with at least two children over six years of age were initially recruited and followed up for 10 years through evaluation visits every 5 years (visit 2 and 3). All subjects were of European-Caucasian origin. Exclusion criteria included the presence of chronic disorders (such as cardiovascular diseases (CVD) or cancer) and an individual history of CVD, thus the individuals are considered as supposed healthy. The study protocol was approved by the local ethics committee and all participants gave their informed written consent. This population is available at the biological resource centre IGE-PCV “Gene-environment interactions in cardiovascular pathophysiology” - BB-0033-00051. Plasma IL6 levels were measured in 853 individuals from the visit 2. The final sample that was included in this project included 757 individuals. This project was co-funded by the European Union within the framework of the Operational Program FEDER-FSE Lorraine et Massif des Vosges 2014-2020 entitled “Gene environment interactions and personalized medicine for cardiovascular pathologies”.

## **2.12 Whitehall II (49, 50)** The Whitehall I study recruited 10308 individuals (67% Male) from 20 civil service departments in London from 1985 to 1988 (Phase 1), of whom 9162 (88.9%) were Caucasians. Participants were aged between 35-55 at Phase 1, and between 56 and 67 at phase 7. Surveys consisted of postal questionnaires in 1989 (Phase 2), 1991-1993 (Phase 3), 1995 (Phase 4), 1997-1999 (Phase 5), 2001 (Phase 6), and 2003-2004 (Phase 7). Clinical examinations were conducted at Phases 1, 3, 5, 7 and full details were reported elsewhere (PMID: **15576467**). Genotyping was performed at the UCL Genomics and Cambridge center, and Illumina Metabochip was used. 2959 individuals with IL6 measures and genotype data participated in the current study.

**S3. Statistical modeling and Quality Control (QC):**

**S3.1 Phenotype trait transformation:**

IL-6 sample measures were first natural log-transformed after which samples with skewed IL-6 levels (mean ± 4 standard deviations) were excluded, to generate an approximately normal distribution.

**S3.2 Phenotype data QC** **(IL6 assay):**

Cohorts with over 5% of their samples measured below the limit of detection underwent a survival-based association analysis as implemented in the lodGWAS package (54). We found no differences in the characteristics of the excluded sample with those of included, except the level of IL-6 was lower in excluded cohorts, due to the issue of a higher number of low measured samples (below detection limit). The reason for IL6 measures below the detection limit in the excluded cohorts is that they have used a different assay (method) than the majority of the included cohorts.

**S3.3 Genotype data QC:**

Quality control of GWAS results files before meta-analyses were carried out using the QCGWAS package in R (51), which performs an automated check of an extended number of quality indicators using statistical inference. The parameter distributions evaluate missing and invalid data, compares the alleles and allele frequencies to a reference panel (HapMap Phase II), compares observed with expected p-values based on beta and SE, and creates skewness and kurtosis graphs, precision plots, histograms, and QQ and Manhattan plots. Using the QCGWAS result files, cohort-specific filter thresholds for the allele frequency and imputation quality were determined if needed to normalize the inflation of statistical tests, else we did not filter for allele frequency, and for imputation quality, instead used method-specific thresholds. An overall summary of QC filters applied in GWAS meta-analysis are shown in Supplementary Figure 1.

**Correction for *ABO* signal:**

An additional QC measure in the genetic association testing comprised, filtering a known false positive GWAS signal (*ABO* gene) while using a specific cytokine measurement kit. Aware of the potential false-positive association observed in previous cytokine GWAS studies (52, 53), in the *ABO* region on chromosome 9, only when an R&D systems high-sensitivity assay kit (R&D system, Minneapolis, MN, USA) was used, we identified the study cohorts using the same kit. Later, the 4 study cohorts (ALSPAC, MONICA/KORA, NTR, and SardiNIA) who identified genome-wide significant associations in the *ABO* region, were asked to rerun the GWAS analysis conditional on the top SNP (rs8176704) for *ABO* locus. None of the other 22 cohorts found this region (*ABO*) genome-wide significant (individually or combined). These four cohorts conditioned their results on their relevant top-SNP in *ABO* (trait ~ snp + covariates + *ABO* snp), the results of which were included in the final discovery meta-analyses.

**S3.4 Meta-analyses:**

Discovery meta-analyses comprising 26 cohorts was conducted using the inverse variance fixed-effects weighted method as implemented in the GWAMA tool (55). Double genomic control (GC) correction was applied for population stratification. For the replication set comprising 12 independent SNPs from 12 different studies, a fixed-effect sample-size weighted Z-score based meta-analysis was conducted using the METAL package (56). The association results from the discovery and replication were also combined using a sample-size weighted Z-score meta-analysis. Variants that were significant in the replication meta-analysis (p<0.05) and that had a p<5×10^-8^ in the combined analysis of the discovery and replication studies with a lower p-value than in the discovery meta-analysis were considered genome-wide significant.

**S4. CHARGE IL-6 GWAS Analysis Plan**

**Project Description:** To conduct a meta-analysis of GWAS data concerning circulating Interleukin-6 (IL-6), a key regulator of the inflammatory response, among participants in several prospective cohort studies.

**Major Phenotypes to Analyze:** ln(IL-6 level) at, or after, the time of DNA collection.

**Participants:** Those with available GWAS genotyping and IL-6 were measured after DNA collection. European ancestry and African-American participants will be analyzed separately.

**Analysis model:** Each cohort will analyze the association of SNPs modeled on an additive/allele dosage scale and ln-transformed IL-6 levels using the set of observed and imputed genotypes (~2.5 million autosomal SNPs). The primary analysis will be adjusted for age and sex. Multi-center studies will additionally adjust for the site; multi-generational studies will additionally adjust for the cohort.

ln (IL-6) ~ SNP + age + sex + study specific variables, as needed

Secondary analyses (to be determined based on results) may consider additional adjustment variables to improve the precision of the outcome variable including smoking, BMI, HRT.

Results from participating cohorts will be combined in a fixed-effects meta-analysis of betas and standard errors. SNPs with a p-value of 1/2.5 million (or one expected false positive), will be considered statistically significant.

**Results Sharing Format** A plain text file with the following columns (please use exact headers, with the following capitalization).

| **variable name** | **description** |
| --- | --- |
| **SNPID** | SNP ID as rs number |
| **Chr** | chromosome number. Use numbers 23 (X), 24 (XY), 25(Y) and 26 (mt) for non-autosomal markers. |
| **position** | physical position for the reference sequence (indicate build 35/36 in readme file) |
| **coded_all** | coded allele also called modeled allele (in the example of A/G SNP in which AA=0, AG=1, and GG=2, the coded allele is G) |
| **noncoded_all** | the other allele |
| **strand_genome** | + or -, representing either the positive/forward strand or the negative/reverse strand of the human genome reference sequence; to clarify which strand the coded_all and noncoded_all are on |
| **beta** | beta estimate from the genotype-phenotype association, at least 5 decimal places -- “NA” if not available |
| **SE** | the standard error of beta estimate, to at least 5 decimal places -- “NA” if not available |
| **pval** | p-value of the test statistic, here just as a double-check -- “NA” if not available |
| **AF_coded_all** | allele frequency for the coded allele -- “NA” if not available |
| **n_total** | total sample with phenotype and genotype for SNP |
| **imputed** | 1/0 coding; 1=imputed SNP, 0=if directly typed |
| **used_for_imp** | 1/0 coding; 1=used for imputation, 0=not used for imputation |
| **oevar_imp** | observed divided by expected variance for imputed allele dosage |

**File Names:** Please choose a filename that begins with your study’s name so that it is easily identifiable. If you are uploading both European Ancestry and African-American results, please indicate this in the filename with EA or AA, respectively.

**Protocol for file sharing:** For the purposes of meta-analysis, sharing will be on the level of analytic results in a format specified by the phenotype working group (see [Results Sharing Format](http://depts.washington.edu/chargeco/wiki/ResultsSharingFormat)). Many working groups use [ShareSpaces](http://catalyst.washington.edu/web_tools/sharespaces.html), a secure web-based file-sharing system implemented by the University of Washington's Catalyst computing group.

**Supplementary Figures**

**Supplementary Figure 1.** Study design overview

**
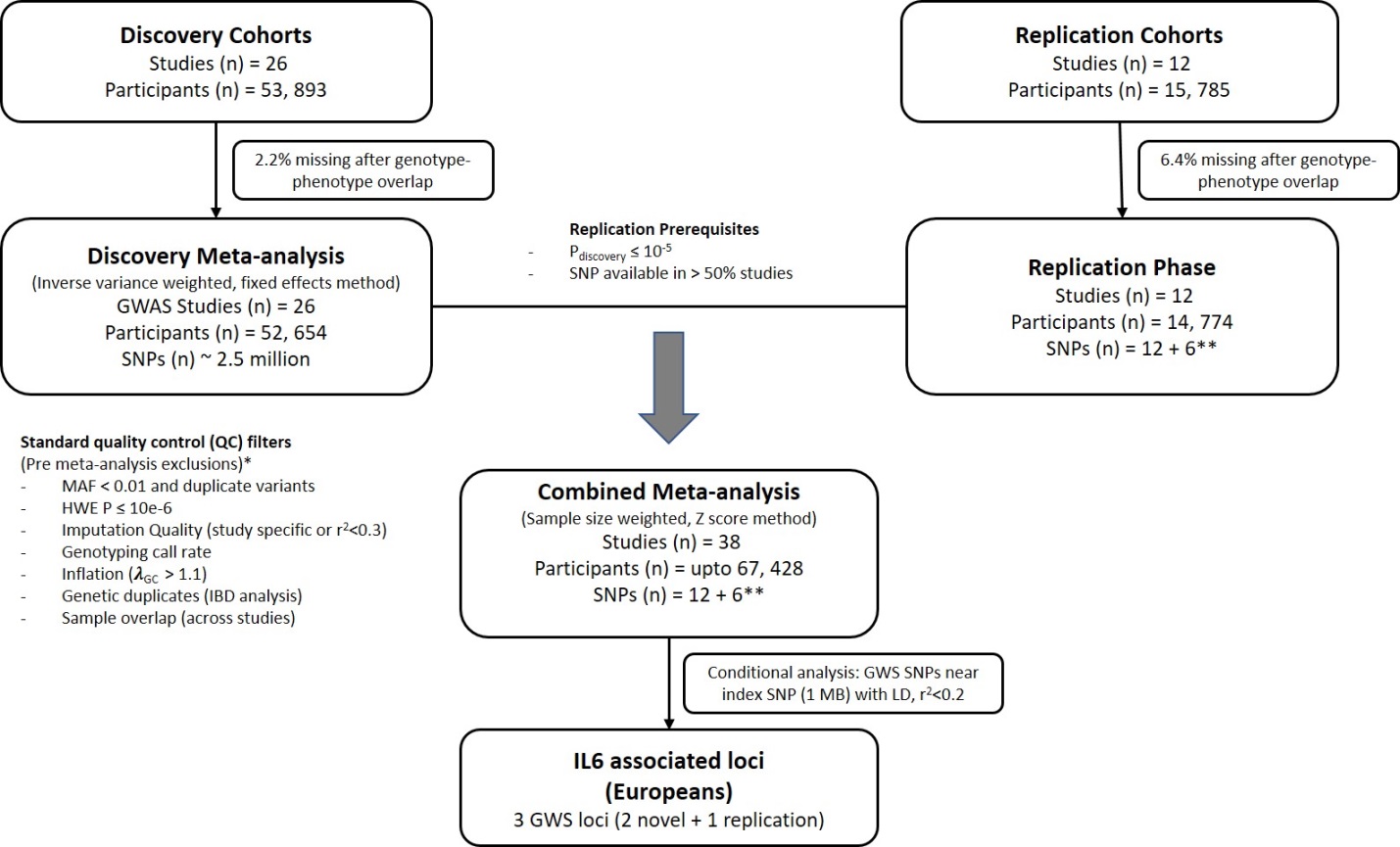
**

GWS: Genome-wide significant (*p*<5×10^-8^); LD: Linkage disequilibrium; *Automated option, QCGWAS R package. ** The additional 6 SNPs include 3 *IL6R* proxy SNPs (LD, r^2^>0.25 with index SNP) and 3 negative controls. Study participants for the discovery and replication stages shown here (52,654 and 14,774) were the final numbers available after combining genotype and phenotype data from the initial study numbers (53,893 and 15, 785). The conditional analysis was performed after confirming results from the combined meta-analysis using genome-wide summary statistics from the discovery meta-analysis.

**Figure 2.1 Forest plot for rs6734238 in the replication stage**


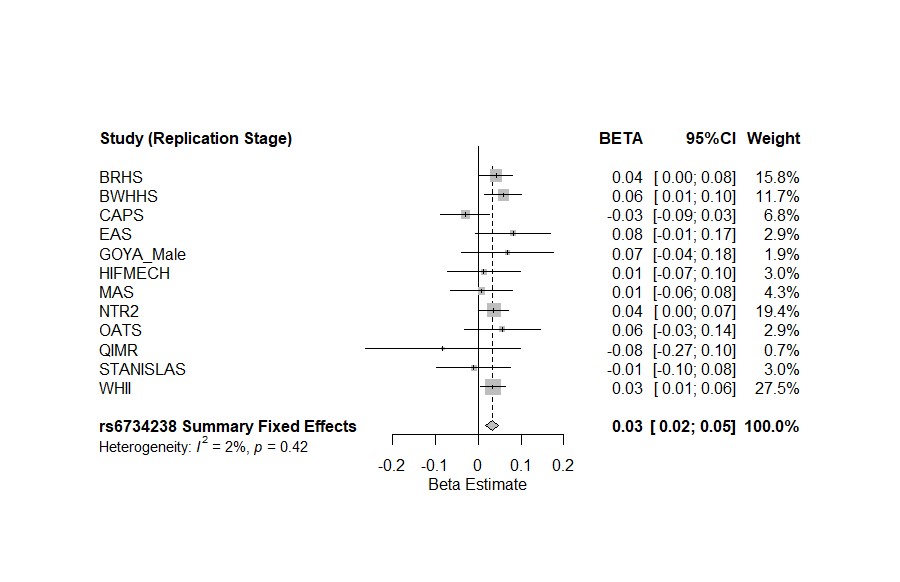


Results correspond Fixed effects inverse variance meta-analyses. I^2^: Heterogeneity (%), p: heterogeneity p value

**Figure 2.2 Forest plot for rs660895 in the replication stage**


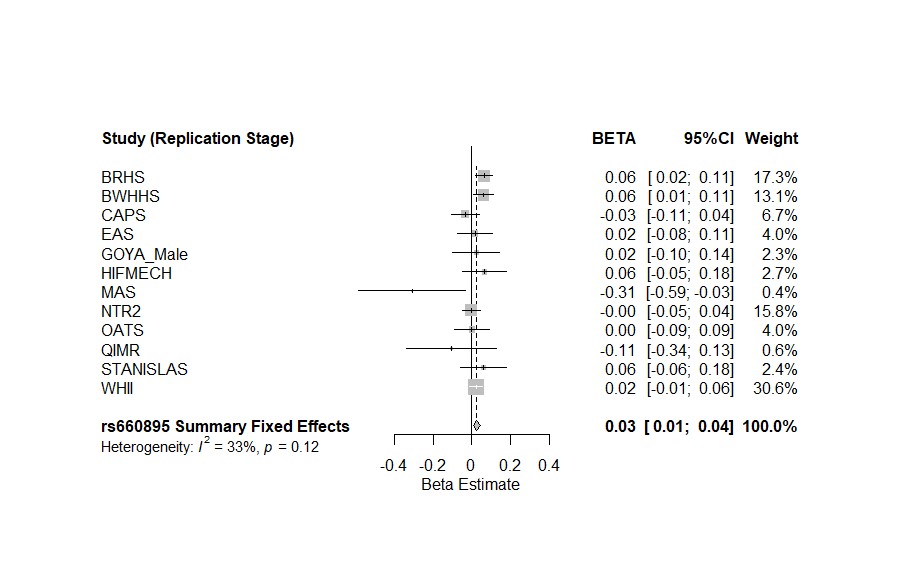


Results correspond to Fixed effects inverse variance meta-analyses. I^2^: Heterogeneity (%), p: heterogeneity p-value

**Figure 2.3 Forest plot for rs4537545 in the replication stage**

**
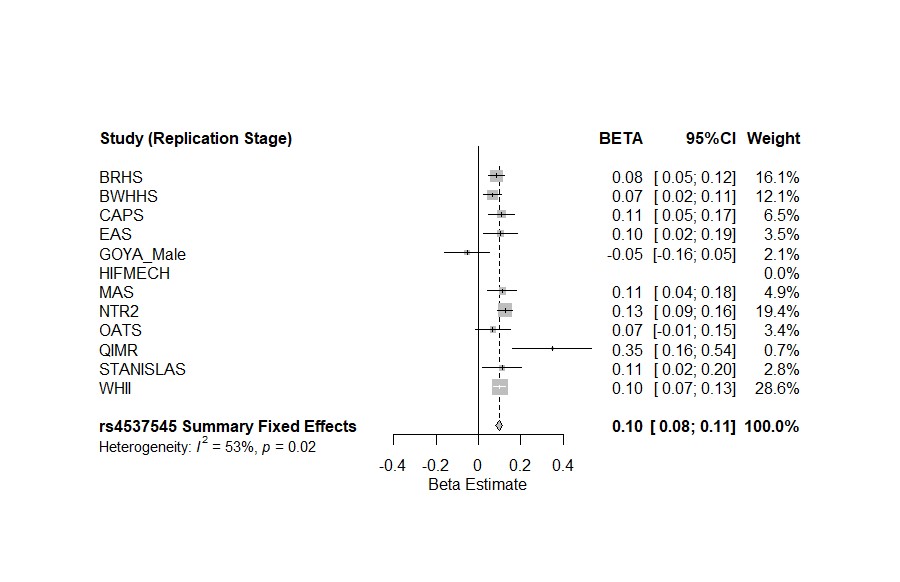
**

Results correspond to Fixed effects inverse variance meta-analyses. I^2^: Heterogeneity (%), p: heterogeneity p-value

#

#

#

#

#

#

#

# **References**

1 Boyd, A., Golding, J., Macleod, J., Lawlor, D.A., Fraser, A., Henderson, J., Molloy, L., Ness, A., Ring, S. and Davey Smith, G. (2013) Cohort Profile: the 'children of the 90s'--the index offspring of the Avon Longitudinal Study of Parents and Children. *Int J Epidemiol*, **42**, 111-127.

2 Mitchell, B.D., McArdle, P.F., Shen, H., Rampersaud, E., Pollin, T.I., Bielak, L.F., Jaquish, C., Douglas, J.A., Roy-Gagnon, M.H., Sack, P. *et al.* (2008) The genetic response to short-term interventions affecting cardiovascular function: rationale and design of the Heredity and Phenotype Intervention (HAPI) Heart Study. *Am Heart J*, **155**, 823-828.

3 Negi, S.I., Pankow, J.S., Fernstrom, K., Hoogeveen, R.C., Zhu, N., Couper, D., Schmidt, M.I., Duncan, B.B. and Ballantyne, C.M. (2012) Racial differences in association of elevated interleukin-18 levels with type 2 diabetes: the Atherosclerosis Risk in Communities study. *Diabetes Care*, **35**, 1513-1518.

4 (1989) The Atherosclerosis Risk in Communities (ARIC) Study: design and objectives. The ARIC investigators. *Am J Epidemiol*, **129**, 687-702.

5 Stenholm, S., Metter, E.J., Roth, G.S., Ingram, D.K., Mattison, J.A., Taub, D.D. and Ferrucci, L. (2011) Relationship between plasma ghrelin, insulin, leptin, interleukin 6, adiponectin, testosterone and longevity in the Baltimore Longitudinal Study of Aging. *Aging Clin Exp Res*, **23**, 153-158.

6 Price, A.L., Patterson, N.J., Plenge, R.M., Weinblatt, M.E., Shadick, N.A. and Reich, D. (2006) Principal components analysis corrects for stratification in genome-wide association studies. *Nat Genet*, **38**, 904-909.

7 Howie, B., Fuchsberger, C., Stephens, M., Marchini, J. and Abecasis, G.R. (2012) Fast and accurate genotype imputation in genome-wide association studies through pre-phasing. *Nat Genet*, **44**, 955-959.

8 Fried, L.P., Borhani, N.O., Enright, P., Furberg, C.D., Gardin, J.M., Kronmal, R.A., Kuller, L.H., Manolio, T.A., Mittelmark, M.B., Newman, A. *et al.* (1991) The Cardiovascular Health Study: design and rationale. *Ann Epidemiol*, **1**, 263-276.

9 Firmann, M., Mayor, V., Vidal, P.M., Bochud, M., Pecoud, A., Hayoz, D., Paccaud, F., Preisig, M., Song, K.S., Yuan, X. *et al.* (2008) The CoLaus study: a population-based study to investigate the epidemiology and genetic determinants of cardiovascular risk factors and metabolic syndrome. *BMC Cardiovasc Disord*, **8**, 6.

10 Higgins, M., Province, M., Heiss, G., Eckfeldt, J., Ellison, R.C., Folsom, A.R., Rao, D.C., Sprafka, J.M. and Williams, R. (1996) NHLBI Family Heart Study: objectives and design. *Am J Epidemiol*, **143**, 1219-1228.

11 Dawber, T.R., Kannel, W.B. and Lyell, L.P. (1963) An approach to longitudinal studies in a community: the Framingham Study. *Ann N Y Acad Sci*, **107**, 539-556.

12 Feinleib, M., Kannel, W.B., Garrison, R.J., McNamara, P.M. and Castelli, W.P. (1975) The Framingham Offspring Study. Design and preliminary data. *Prev Med*, **4**, 518-525.

13 Splansky, G.L., Corey, D., Yang, Q., Atwood, L.D., Cupples, L.A., Benjamin, E.J., D'Agostino, R.B., Sr., Fox, C.S., Larson, M.G., Murabito, J.M. *et al.* (2007) The Third Generation Cohort of the National Heart, Lung, and Blood Institute's Framingham Heart Study: design, recruitment, and initial examination. *Am J Epidemiol*, **165**, 1328-1335.

14 Corella, D., Arnett, D.K., Tsai, M.Y., Kabagambe, E.K., Peacock, J.M., Hixson, J.E., Straka, R.J., Province, M., Lai, C.Q., Parnell, L.D. *et al.* (2007) The -256T>C polymorphism in the apolipoprotein A-II gene promoter is associated with body mass index and food intake in the genetics of lipid lowering drugs and diet network study. *Clin Chem*, **53**, 1144-1152.

15 Barker, D.J., Osmond, C., Forsen, T.J., Kajantie, E. and Eriksson, J.G. (2005) Trajectories of growth among children who have coronary events as adults. *N Engl J Med*, **353**, 1802-1809.

16 Eriksson, J.G., Osmond, C., Kajantie, E., Forsen, T.J. and Barker, D.J. (2006) Patterns of growth among children who later develop type 2 diabetes or its risk factors. *Diabetologia*, **49**, 2853-2858.

17 Kalogeropoulos, A., Georgiopoulou, V., Psaty, B.M., Rodondi, N., Smith, A.L., Harrison, D.G., Liu, Y., Hoffmann, U., Bauer, D.C., Newman, A.B. *et al.* (2010) Inflammatory markers and incident heart failure risk in older adults: the Health ABC (Health, Aging, and Body Composition) study. *J Am Coll Cardiol*, **55**, 2129-2137.

18 Ferrucci, L., Bandinelli, S., Benvenuti, E., Di Iorio, A., Macchi, C., Harris, T.B. and Guralnik, J.M. (2000) Subsystems contributing to the decline in ability to walk: bridging the gap between epidemiology and geriatric practice in the InCHIANTI study. *J Am Geriatr Soc*, **48**, 1618-1625.

19 Deary, I.J., Whiteman, M.C., Starr, J.M., Whalley, L.J. and Fox, H.C. (2004) The impact of childhood intelligence on later life: following up the Scottish mental surveys of 1932 and 1947. *J Pers Soc Psychol*, **86**, 130-147.

20 Deary, I.J., Gow, A.J., Taylor, M.D., Corley, J., Brett, C., Wilson, V., Campbell, H., Whalley, L.J., Visscher, P.M., Porteous, D.J. *et al.* (2007) The Lothian Birth Cohort 1936: a study to examine influences on cognitive ageing from age 11 to age 70 and beyond. *BMC Geriatr*, **7**, 28.

21 Deary, I.J., Gow, A.J., Pattie, A. and Starr, J.M. (2012) Cohort profile: the Lothian Birth Cohorts of 1921 and 1936. *Int J Epidemiol*, **41**, 1576-1584.

22 van der Spoel, E., Rozing, M.P., Houwing-Duistermaat, J.J., Slagboom, P.E., Beekman, M., de Craen, A.J., Westendorp, R.G. and van Heemst, D. (2015) Association analysis of insulin-like growth factor-1 axis parameters with survival and functional status in nonagenarians of the Leiden Longevity Study. *Aging (Albany NY)*, **7**, 956-963.

23 Nauck, M., Winkelmann, B.R., Hoffmann, M.M., Bohm, B.O., Wieland, H. and Marz, W. (2002) The interleukin-6 G(-174)C promoter polymorphism in the LURIC cohort: no association with plasma interleukin-6, coronary artery disease, and myocardial infarction. *J Mol Med (Berl)*, **80**, 507-513.

24 Koenig, W., Khuseyinova, N., Baumert, J., Thorand, B., Loewel, H., Chambless, L., Meisinger, C., Schneider, A., Martin, S., Kolb, H. *et al.* (2006) Increased concentrations of C-reactive protein and IL-6 but not IL-18 are independently associated with incident coronary events in middle-aged men and women: results from the MONICA/KORA Augsburg case-cohort study, 1984-2002. *Arterioscler Thromb Vasc Biol*, **26**, 2745-2751.

25 Andersson, N., Strandberg, L., Nilsson, S., Adamovic, S., Karlsson, M.K., Ljunggren, O., Mellstrom, D., Lane, N.E., Zmuda, J.M., Nielsen, C. *et al.* (2010) A variant near the interleukin-6 gene is associated with fat mass in Caucasian men. *Int J Obes (Lond)*, **34**, 1011-1019.

26 Prather, A.A., Vogelzangs, N. and Penninx, B.W. (2015) Sleep duration, insomnia, and markers of systemic inflammation: results from the Netherlands Study of Depression and Anxiety (NESDA). *J Psychiatr Res*, **60**, 95-102.

27 Willemsen, G., de Geus, E.J., Bartels, M., van Beijsterveldt, C.E., Brooks, A.I., Estourgie-van Burk, G.F., Fugman, D.A., Hoekstra, C., Hottenga, J.J., Kluft, K. *et al.* (2010) The Netherlands Twin Register biobank: a resource for genetic epidemiological studies. *Twin Res Hum Genet*, **13**, 231-245.

28 Postmus, I., Johnson, P.C., Trompet, S., de Craen, A.J., Slagboom, P.E., Devlin, J.J., Shiffman, D., Sacks, F.M., Kearney, P.M., Stott, D.J. *et al.* (2014) In search for genetic determinants of clinically meaningful differential cardiovascular event reduction by pravastatin in the PHArmacogenetic study of Statins in the Elderly at risk (PHASE)/PROSPER study. *Atherosclerosis*, **235**, 58-64.

29 Hofman, A., Brusselle, G.G., Darwish Murad, S., van Duijn, C.M., Franco, O.H., Goedegebure, A., Ikram, M.A., Klaver, C.C., Nijsten, T.E., Peeters, R.P. *et al.* (2015) The Rotterdam Study: 2016 objectives and design update. *Eur J Epidemiol*, **30**, 661-708.

30 Pani, A., Bragg-Gresham, J., Masala, M., Piras, D., Atzeni, A., Pilia, M.G., Ferreli, L., Balaci, L., Curreli, N., Delitala, A. *et al.* (2014) Prevalence of CKD and its relationship to eGFR-related genetic loci and clinical risk factors in the SardiNIA study cohort. *J Am Soc Nephrol*, **25**, 1533-1544.

31 John, U., Greiner, B., Hensel, E., Ludemann, J., Piek, M., Sauer, S., Adam, C., Born, G., Alte, D., Greiser, E. *et al.* (2001) Study of Health In Pomerania (SHIP): a health examination survey in an east German region: objectives and design. *Soz Praventivmed*, **46**, 186-194.

32 Volzke, H., Alte, D., Schmidt, C.O., Radke, D., Lorbeer, R., Friedrich, N., Aumann, N., Lau, K., Piontek, M., Born, G. *et al.* (2011) Cohort profile: the study of health in Pomerania. *Int J Epidemiol*, **40**, 294-307.

33 Sas, A.A., Jamshidi, Y., Zheng, D., Wu, T., Korf, J., Alizadeh, B.Z., Spector, T.D. and Snieder, H. (2012) The age-dependency of genetic and environmental influences on serum cytokine levels: a twin study. *Cytokine*, **60**, 108-113.

34 Hulkkonen, J., Lehtimaki, T., Mononen, N., Juonala, M., Hutri-Kahonen, N., Taittonen, L., Marniemi, J., Nieminen, T., Viikari, J., Raitakari, O. *et al.* (2009) Polymorphism in the IL6 promoter region is associated with the risk factors and markers of subclinical atherosclerosis in men: The Cardiovascular Risk in Young Finns Study. *Atherosclerosis*, **203**, 454-458.

35 Shaper, A.G., Pocock, S.J., Walker, M., Cohen, N.M., Wale, C.J. and Thomson, A.G. (1981) British Regional Heart Study: cardiovascular risk factors in middle-aged men in 24 towns. *Br Med J (Clin Res Ed)*, **283**, 179-186.

36 Walker, M., Whincup, P.H. and Shaper, A.G. (2004) The British Regional Heart Study 1975-2004. *Int J Epidemiol*, **33**, 1185-1192.

37 Lawlor, D.A., Bedford, C., Taylor, M. and Ebrahim, S. (2003) Geographical variation in cardiovascular disease, risk factors, and their control in older women: British Women's Heart and Health Study. *J Epidemiol Community Health*, **57**, 134-140.

38 Fraser, A., May, M., Lowe, G., Rumley, A., Smith, G.D., Ebrahim, S. and Lawlor, D.A. (2009) Interleukin-6 and incident coronary heart disease: results from the British Women's Heart and Health Study. *Atherosclerosis*, **202**, 567-572.

39 Patterson, C.C., Smith, A.E., Yarnell, J.W., Rumley, A., Ben-Shlomo, Y. and Lowe, G.D. (2010) The associations of interleukin-6 (IL-6) and downstream inflammatory markers with risk of cardiovascular disease: the Caerphilly Study. *Atherosclerosis*, **209**, 551-557.

40 Shah, T., Engmann, J., Dale, C., Shah, S., White, J., Giambartolomei, C., McLachlan, S., Zabaneh, D., Cavadino, A., Finan, C. *et al.* (2013) Population genomics of cardiometabolic traits: design of the University College London-London School of Hygiene and Tropical Medicine-Edinburgh-Bristol (UCLEB) Consortium. *PLoS One*, **8**, e71345.

41 Fowkes, F.G., Housley, E., Cawood, E.H., Macintyre, C.C., Ruckley, C.V. and Prescott, R.J. (1991) Edinburgh Artery Study: prevalence of asymptomatic and symptomatic peripheral arterial disease in the general population. *Int J Epidemiol*, **20**, 384-392.

42 Paternoster, L., Evans, D.M., Nohr, E.A., Holst, C., Gaborieau, V., Brennan, P., Gjesing, A.P., Grarup, N., Witte, D.R., Jorgensen, T. *et al.* (2011) Genome-wide population-based association study of extremely overweight young adults--the GOYA study. *PLoS One*, **6**, e24303.

43 Hollensted, M., Ahluwalia, T.S., Have, C.T., Grarup, N., Fonvig, C.E., Nielsen, T.R., Trier, C., Paternoster, L., Pedersen, O., Holm, J.C. *et al.* (2015) Common variants in LEPR, IL6, AMD1, and NAMPT do not associate with risk of juvenile and childhood obesity in Danes: a case-control study. *BMC Med Genet*, **16**, 105.

44 Kelberman, D., Hawe, E., Luong, L.A., Mohamed-Ali, V., Lundman, P., Tornvall, P., Aillaud, M.F., Juhan-Vague, I., Yudkin, J.S., Margaglione, M. *et al.* (2004) Effect of Interleukin-6 promoter polymorphisms in survivors of myocardial infarction and matched controls in the North and South of Europe. The HIFMECH Study. *Thromb Haemost*, **92**, 1122-1128.

45 Sachdev, P.S., Brodaty, H., Reppermund, S., Kochan, N.A., Trollor, J.N., Draper, B., Slavin, M.J., Crawford, J., Kang, K., Broe, G.A. *et al.* (2010) The Sydney Memory and Ageing Study (MAS): methodology and baseline medical and neuropsychiatric characteristics of an elderly epidemiological non-demented cohort of Australians aged 70-90 years. *Int Psychogeriatr*, **22**, 1248-1264.

46 Sachdev, P.S., Lammel, A., Trollor, J.N., Lee, T., Wright, M.J., Ames, D., Wen, W., Martin, N.G., Brodaty, H., Schofield, P.R. *et al.* (2009) A comprehensive neuropsychiatric study of elderly twins: the Older Australian Twins Study. *Twin Res Hum Genet*, **12**, 573-582.

47 Revez, J.A., Bain, L., Chapman, B., Powell, J.E., Jansen, R., Duffy, D.L., Tung, J.Y., Collaborators, A., Penninx, B.W., Visscher, P.M. *et al.* (2013) A new regulatory variant in the interleukin-6 receptor gene associates with asthma risk. *Genes Immun*, **14**, 441-446.

48 Visvikis-Siest, S. and Siest, G. (2008) The STANISLAS Cohort: a 10-year follow-up of supposed healthy families. Gene-environment interactions, reference values and evaluation of biomarkers in prevention of cardiovascular diseases. *Clin Chem Lab Med*, **46**, 733-747.

49 Marmot, M. and Brunner, E. (2005) Cohort Profile: the Whitehall II study. *Int J Epidemiol*, **34**, 251-256.

50 Sanderson, S.C., Kumari, M., Brunner, E.J., Miller, M.A., Rumley, A., Lowe, G.D., Marmot, M.G. and Humphries, S.E. (2009) Association between IL6 gene variants -174G>C and -572G>C and serum IL-6 levels: interactions with social position in the Whitehall II cohort. *Atherosclerosis*, **204**, 459-464.

51 van der Most, P.J., Vaez, A., Prins, B.P., Munoz, M.L., Snieder, H., Alizadeh, B.Z. and Nolte, I.M. (2014) QCGWAS: A flexible R package for automated quality control of genome-wide association results. *Bioinformatics*, **30**, 1185-1186.

52 Melzer, D., Perry, J.R., Hernandez, D., Corsi, A.M., Stevens, K., Rafferty, I., Lauretani, F., Murray, A., Gibbs, J.R., Paolisso, G. *et al.* (2008) A genome-wide association study identifies protein quantitative trait loci (pQTLs). *PLoS Genet*, **4**, e1000072.

53 Neijts, M., van Dongen, J., Kluft, C., Boomsma, D.I., Willemsen, G. and de Geus, E.J. (2013) Genetic architecture of the pro-inflammatory state in an extended twin-family design. *Twin Res Hum Genet*, **16**, 931-940.

54 Vaez, A., van der Most, P.J., Prins, B.P., Snieder, H., van den Heuvel, E., Alizadeh, B.Z. and Nolte, I.M. (2016) lodGWAS: a software package for genome-wide association analysis of biomarkers with a limit of detection. *Bioinformatics*, **32**, 1552-1554.

55 Magi, R. and Morris, A.P. (2010) GWAMA: software for genome-wide association meta-analysis. *BMC Bioinformatics*, **11**, 288.

56 Willer, C.J., Li, Y. and Abecasis, G.R. (2010) METAL: fast and efficient meta-analysis of genomewide association scans. *Bioinformatics*, **26**, 2190-2191.
